# Crucial role of iron in epigenetic rewriting during adipocyte differentiation mediated by JMJD1A and TET2 activity

**DOI:** 10.1093/nar/gkad342

**Published:** 2023-05-09

**Authors:** Tomohiro Suzuki, Tetsuro Komatsu, Hiroshi Shibata, Akiko Tanioka, Diana Vargas, Reika Kawabata-Iwakawa, Fumihito Miura, Shinnosuke Masuda, Mayuko Hayashi, Kyoko Tanimura-Inagaki, Sumiyo Morita, Junki Kohmaru, Koji Adachi, Masayuki Tobo, Hideru Obinata, Tasuku Hirayama, Hiroshi Kimura, Juro Sakai, Hideko Nagasawa, Hideyuki Itabashi, Izuho Hatada, Takashi Ito, Takeshi Inagaki

**Affiliations:** Laboratory of Epigenetics and Metabolism, Institute for Molecular and Cellular Regulation, Gunma University, Gunma371-8512, Japan; Laboratory of Epigenetics and Metabolism, Institute for Molecular and Cellular Regulation, Gunma University, Gunma371-8512, Japan; Laboratory of Epigenetics and Metabolism, Institute for Molecular and Cellular Regulation, Gunma University, Gunma371-8512, Japan; Laboratory of Epigenetics and Metabolism, Institute for Molecular and Cellular Regulation, Gunma University, Gunma371-8512, Japan; Laboratory of Epigenetics and Metabolism, Institute for Molecular and Cellular Regulation, Gunma University, Gunma371-8512, Japan; Division of Integrated Oncology Research, Gunma University Initiative for Advanced Research, Gunma University, Gunma371-8511, Japan; Department of Biochemistry, Kyushu University Graduate School of Medical Sciences, Fukuoka 812-8582, Japan; Laboratory of Epigenetics and Metabolism, Institute for Molecular and Cellular Regulation, Gunma University, Gunma371-8512, Japan; Laboratory of Epigenetics and Metabolism, Institute for Molecular and Cellular Regulation, Gunma University, Gunma371-8512, Japan; Laboratory of Epigenetics and Metabolism, Institute for Molecular and Cellular Regulation, Gunma University, Gunma371-8512, Japan; Department of Endocrinology, Metabolism and Nephrology, Graduate School of Medicine, Nippon Medical School, Tokyo 113-8602, Japan; Laboratory of Genome Science, Biosignal Genome Resource Center, Institute for Molecular and Cellular Regulation, Gunma University, Gunma371-8512, Japan; Institute for Molecular and Cellular Regulation Joint Usage/Research Support Center, Gunma University, Gunma371-8512, Japan; Kaihin Makuhari Laboratory, PerkinElmer Japan Co., Ltd., Chiba261-8501, Japan; Institute for Molecular and Cellular Regulation Joint Usage/Research Support Center, Gunma University, Gunma371-8512, Japan; Education and Research Support Center, Gunma University Graduate School of Medicine, Gunma371-8511, Japan; Laboratory of Pharmaceutical and Medicinal Chemistry, Gifu Pharmaceutical University, Gifu501-1196, Japan; Cell Biology Center, Tokyo Institute of Technology, Kanagawa226-8503, Japan; Division of Metabolic Medicine, Research Center for Advanced Science and Technology, The University of Tokyo, Tokyo153-8904, Japan; Division of Molecular Physiology and Metabolism, Tohoku University Graduate School of Medicine, Sendai 980-8575, Japan; Laboratory of Pharmaceutical and Medicinal Chemistry, Gifu Pharmaceutical University, Gifu501-1196, Japan; Graduate School of Science and Technology, Gunma University, Gunma376-8515, Japan; Laboratory of Genome Science, Biosignal Genome Resource Center, Institute for Molecular and Cellular Regulation, Gunma University, Gunma371-8512, Japan; Viral Vector Core, Gunma University Initiative for Advanced Research, Gunma371-8511, Japan; Department of Biochemistry, Kyushu University Graduate School of Medical Sciences, Fukuoka 812-8582, Japan; Laboratory of Epigenetics and Metabolism, Institute for Molecular and Cellular Regulation, Gunma University, Gunma371-8512, Japan

## Abstract

Iron metabolism is closely associated with the pathogenesis of obesity. However, the mechanism of the iron-dependent regulation of adipocyte differentiation remains unclear. Here, we show that iron is essential for rewriting of epigenetic marks during adipocyte differentiation. Iron supply through lysosome-mediated ferritinophagy was found to be crucial during the early stage of adipocyte differentiation, and iron deficiency during this period suppressed subsequent terminal differentiation. This was associated with demethylation of both repressive histone marks and DNA in the genomic regions of adipocyte differentiation-associated genes, 　including *Pparg*, which encodes PPARγ, the master regulator of adipocyte differentiation. In addition, we identified several epigenetic demethylases to be responsible for iron-dependent adipocyte differentiation, with the histone demethylase jumonji domain-containing 1A and the DNA demethylase ten-eleven translocation 2 as the major enzymes. The interrelationship between repressive histone marks and DNA methylation was indicated by an integrated genome-wide association analysis, and was also supported by the findings that both histone and DNA demethylation were suppressed by either the inhibition of lysosomal ferritin flux or the knockdown of iron chaperone poly(rC)-binding protein 2. In summary, epigenetic regulations through iron-dependent control of epigenetic enzyme activities play an important role in the organized gene expression mechanisms of adipogenesis.

## Graphical Abstract

**Graphical Abstract:**
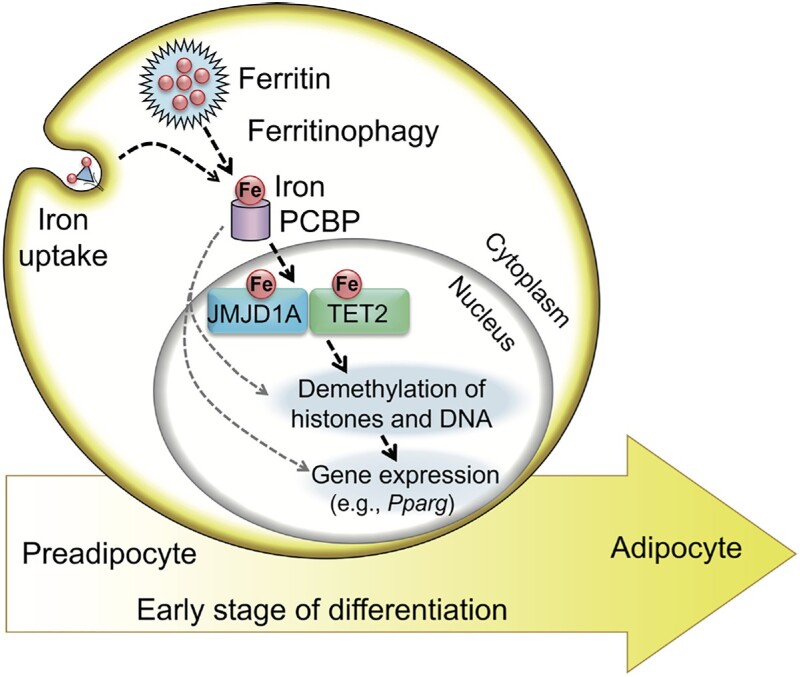
Crucial role of iron in epigenetic rewriting during adipocyte differentiation mediated by JMJD1A and TET2 activity.

## INTRODUCTION

Iron is closely associated with the pathophysiology of obesity (1). For example, a low-iron diet as well as administration of iron chelators significantly prevent diabetes in rodent models of obesity (2). It has also been reported that iron chelators inhibit adipogenesis (3–5). However, the mechanism underlying the iron-obesity interrelationship is complex, because whole-body iron homeostasis is affected by the systemic crosstalk of multiple organs. Therefore, elucidation of the cell-autonomous molecular actions of iron is required to understand the iron-dependent molecular mechanisms of obesity.

Iron is an essential metal that is required for a variety of biological processes, including fuel oxidation and electron transport in mitochondria, as well as for the activation of various 2-oxoglutarate-dependent dioxygenases (2OGDs) (6–8). The 2OGDs include the ten-eleven translocation (TET) enzymes, alkB homolog (ALKBH) proteins, and jumonji C (JmjC)-domain containing demethylases, most of which catalyze the demethylation of DNA, RNA and histones, respectively. Thus, iron regulates a wide range of epigenetic mechanisms and is expected to affect transcriptional and post-transcriptional control. Whereas iron is essential for biological processes, it can also be harmful because ferrous iron can induce the production of reactive oxygen species, causing oxidative damage. Therefore, intracellular iron homeostasis needs to be tightly controlled via its sequestration by the iron storage protein ferritin, and its transport in a nontoxic state by iron-binding chaperone proteins, namely, poly(rC)-binding proteins (PCBPs) (6,9). In response to increased iron demand, ferritin is degraded by a selective type of autophagy called ferritinophagy (10). During the process of ferritinophagy, nuclear receptor coactivator 4 (NCOA4) acts as a selective cargo adaptor to target ferritin to phagophores, which are precursors of autophagosomes that eventually fuse with lysosomes (11,12).

Adipocyte differentiation is mediated by a highly organized gene expression program involving a series of transcription factors and epigenetic modifications (13–15). Particularly, peroxisome proliferator-activated receptor γ (PPARγ) and C/CAAT enhancer-binding proteins (C/EBPs) are crucial transcription factors for adipocyte differentiation. For example, during the differentiation process of the 3T3-L1 adipocyte cell line, C/EBPβ is expressed within 4 h after the induction of adipocyte differentiation, and induces *Pparg* and *Cebpa* expression in concert with the epigenetic coordination of DNA methylation and histone modifications, such as the methylation of histones H3K4, H3K9 and H3K27 after a delay of about 12 h (14,16,17). The expression of a series of genes that define terminal differentiation are then induced.

It has been reported that C/EBPβ activates the expression of the autophagy-related gene Atg4b (18), suggesting the possible role of autophagy in adipocyte differentiation. Indeed, multiple reports have shown that autophagy is not only active but also essential in the early stage of adipocyte differentiation (18–21). The suppression of autophagy was closely associated with the downregulation of PPARγ and C/EBPα, and repressed adipocyte differentiation (18,19,22). Thus, autophagy is involved in the transcriptional regulation of adipocyte differentiation. In this study, we show that iron demand is increased in the early stage of adipocyte differentiation, and thus ferritinophagy is induced at this stage. We also show that the subcellular iron depletion through decreased iron supply or the insufficient subcellular iron transport reduces iron-dependent histone demethylation and DNA demethylation, and suppresses the terminal differentiation of adipocytes.

## MATERIALS AND METHODS

### Cell culture

Maintenance and differentiation of 3T3-L1 preadipocytes (provided by Dr. Howard Green) (23) were carried out as described previously (24). Deferoxamine (DFO) (Sigma-Aldrich, D-9533) was freshly diluted in Dulbecco's modified Eagle's medium (DMEM), and added to the culture medium at the indicated concentrations. Stock solutions of PIK-III (Selleck Biotech, S7683), bafilomycin A1 (Cayman Chemical Company, 11038), and 2,2’-bipyridyl (FUJIFILM Wako, 042–04241) were prepared by diluting them in dimethyl sulfide (DMSO) at 10, 1 and 100 mM, respectively and added to the medium at the indicated concentrations, where final DMSO concentrations were 0.01%, 0.01% and 0.02%, respectively. Oil red-O (ORO) staining was performed as described previously (25), and photographed with a GT-X980 scanner (EPSON) and an inverted microscope Primovert (Zeiss). The cell number counting assay was performed at the indicated time points during adipocyte differentiation by detaching cells using 0.25% trypsin (FUJIFILM Wako, 200-13953), 0.75 mg/ml collagenase (FUJIFILM Wako, 17105-041), and 0.5 mg/ml Dispase II (Thermo Fisher, 17105-041) in DMEM.

### Plasmid construction and retroviral transduction

To construct retrovirus-based short hairpin RNA (shRNA) expression vectors for knockdown of epigenetic demethylases, two oligonucleotides containing shRNA sequences (Supplementary Table S1) were annealed and inserted into a mouse U6 promoter-driven vector pMXs-U6 puro (provided by Dr Toshio Kitamura) (26). Expression plasmids of individual enzymes (Supplementary Table S2) were constructed using a long terminal repeat promoter-driven expression vector pMXs-puro (from Dr. T. Kitamura), in which the original puromycin-resistant gene was replaced with the blasticidin S-resistant gene. The iron-binding site of each enzyme sequence was modified by a previously reported mutation by the PCR-based site-directed mutagenesis (27–43) (Supplementary Table S2). Using the similar method, shRNA-resistant plasmids were prepared by introducing silent mutations in the targeted sequence (Supplementary Table S3). Retroviruses were produced by transfecting each plasmid into Platinum-E packaging cells (from Dr T. Kitamura) using polyethyleneimine MAX (Polyscience, 24765-1). Retrovirus infection was performed as reported previously (25) and the infected cells were selected by 10 μg/ml puromycin (Thermo Fisher, ant-pr-1) or 10 μg/ml blasticidin S (Thermo Fisher, ant-bl-1).

### Real-time quantitative PCR (qPCR)

qPCR was performed as described previously (24) using primers in Supplementary Table S4. Data was analyzed by the standard curve method and normalized to the level of *Cyclophilin B* (*Ppib*) as an internal control.

### Immunoblotting

For preparation of whole-cell lysate, cells were washed with PBS and lysed in 1.5× Laemmli SDS sample buffer containing 25 mM DTT and protease inhibitor cocktail (Roche, 05056489001). After boiling at 95°C for 5 min, the sample was sonicated for 5 s × 20 times with 5 s intervals with Bioruptor II, Type 24 (Sonic Bio Co.). The supernatant was collected after centrifugation at 15 000 × g for 15 min at 4°C and was used as the whole-cell lysate. The nuclear fractions were prepared as reported elsewhere with slight modifications (44). Cells on a 60 mm dish were allowed to swell on ice in 0.4 ml of buffer A (10 mM KCl, 1.5 mM MgCl_2_, 1 mM EDTA, 1 mM EGTA, 1 mM DTT, 10 mM HEPES–KOH, pH 7.6, and protease inhibitor cocktail [Roche, 05056489001]), scraped in a 1.5 ml microcentrifuge tube, and centrifuged at 1000 × g at 4°C for 7 min. The precipitate was collected as a nuclear pellet. The nuclear pellet was resuspended in 0.2 ml of buffer B (0.42 M NaCl, 1.5 mM MgCl_2_, 2.5% glycerol, 1 mM EDTA, 1 mM EGTA, 1 mM DTT, 20 mM HEPES–KOH, pH 7.6, and protease inhibitor cocktail [Roche, 05056489001]). After rotation at 4°C for 1 h, the suspension was centrifuged at 10^5^ × g for 30 min at 4°C to collect the supernatant as the nuclear extract. Laemmli SDS sample buffer was added to these fractions at the final 1x concentration, and the samples were boiled at 95°C for 5 min. The protein samples were used for immunoblotting and protein assay using Pierce 660nm Protein Assay Reagent (Thermo Fisher, 22660) with Ionic Detergent Compatibility Reagent (Thermo Fisher, 22663). The same amounts of proteins were separated on a SDS polyacrylamide gel or a 4–20% gradient SDS-polyacrylamide gel (Bio-Rad, 4561096) and transferred onto a polyvinylidene difluoride membrane (Millipore, ISEQ85R) or a nitrocellulose membrane (Bio-Rad, 1620097). Membranes were blocked with 5% skimmed milk or 5% bovine serum albumin in TBS-Tween 20 for 1 h at room temperature (RT) and incubated with primary antibodies at the indicated dilution (Supplementary Table S5) overnight at 4°C or for 1–2 h at RT, followed by an anti-rabbit or anti-mouse horseradish peroxidase (HRP)-conjugated secondary antibody (anti-rabbit: Cell Signaling Technology, #7074, anti-mouse: Sigma-Aldrich, A4416) treatment for 1 h at RT. Alternatively, an anti-H3K9me2 antibody (from Dr. Kimura, Supplementary Table S5) was used after its direct conjugation to HRP using Peroxidase Labeling Kit (Dojindo, LK11). Band signals were detected with Immobilon Crescendo Western HRP substrate (Millipore, WBLUR0500) using LAS-4000 image analyzer (FUJIFILM). Band intensities were quantified with Multi Gauge software (FUJIFILM) or ImageJ/Fiji software. The band intensities on different membranes or gels were adjusted with the average intensity of the common group among them. The intensities of proteins of interest were normalized to those of the indicated internal controls or the whole protein levels. The whole protein levels were measured using 4–20% Mini-PROTEAN TGX Stain-Free gels (Bio-Rad, 4561036), the GelDoc XR+ system (Bio-Rad), and the ImageLab software (Bio-Rad) in the setting of 5 min UV irradiation, and then the intensity values were used as an internal control as described in the guideline for autophagy evaluation (45).

### Measurement of lysosome flux and ferritin degradation rate

Lysosome flux and ferritin degradation rate were measured by the immunoblotting-based method. For lysosome flux analysis, cells were incubated with 100 nM of bafilomycin A1 for 24 h before sample collection on Days 0, 1 and 2. For measurement of ferritin degradation rate, cells on Days 0, 1 and 2 were treated with 200 μM of 2,2’-bipyridyl for 0, 3, 6 and 9 h. The involvement of lysosomal degradation was examined by treating cells simultaneously with 200 μM of 2,2’-bipyridyl and 100 nM of bafilomycin A1 for 3 h on Days 0, 1 and 2. At the end of each drug treatment, the whole cell lysates were prepared and subjected to immunoblotting.

### Immunocytochemistry, signal quantification, and colocalization analysis

For immunocytochemistry, cells were grown and differentiated on a 35 mm glass bottom dish (MatTek, P35G-0-14-C). The cells were treated with 100 nM bafilomycin A1 for 6 h before fixation especially for co-localization analysis. Cell fixation was performed with the solution containing 4% paraformaldehyde, 0.1% Triton X-100 and 200 mM HEPES–NaOH, pH 7.5, for 5 min at RT, permeabilized with 1% Triton X-100 in PBS for 20 min at RT, and blocked with 5% normal goat serum in PBS for 30 min. Primary antibodies were diluted in 1% normal goat serum in PBS, applied to the cells, and incubated either at 4ºC overnight (anti-H3K9me2 [Supplementary Table S5]), at RT for 1 h (anti-H3K9me3, anti-H3K27me3, anti-H3K27ac, anti-PCBP1 and anti-PCBP2 [Supplementary Table S5]), or at RT overnight (anti-LC3, anti-ferritin, and anti-NCOA4 [Supplementary Table S5]). Staining was visualized by incubating the cells with secondary antibodies (Alexa Fluor 488-labeled anti-mouse IgG [1:1000, for anti-H3K9me2; Thermo Fisher, A11029], Alexa Fluor 488-labeled anti-rabbit IgG [1:1000, for anti-LC3, anti-PCBP1, and anti-PCBP2; Thermo Fisher, A11008], and Alexa Fluor 568-labeled anti-mouse IgG [1:1000, for anti-NCOA4 and anti-ferritin; Thermo Fisher, A11031]) in 1% normal goat serum in PBS, except anti-H3K9me3, anti-H3K27me3, and anti-H3K27ac antibodies that were directly conjugated with a green fluorescent dye (Dojindo Laboratories, Supplementary Table S5).

To evaluate H3K9me2 levels in 3T3-L1 preadipocytes electroporated with pCMV-HA-Jmjd1a by GenePulser Xcell (Bio-Rad), cells were fixed, permeabilized, and then stained with anti-H3K9me2 and anti-HA antibodies (Supplementary Table S5) in 1% normal goat serum in PBS for 2 h at RT, followed by double-staining with Alexa Fluor 488-labeled anti-mouse IgG (1:1000) and Alexa Fluor 647-labeled anti-rabbit IgG (1:1000; Thermo Fisher, A31574) in 1% normal goat serum in PBS. To evaluate 5-methyl cytosine (5mC) levels in 3T3-L1 preadipocytes electroporated with pCMV-HA-Tet2, after fixation and permeabilization, cells were treated with 4 N HCl for 10 min at RT, and washed using 0.1 M borate buffer (pH 8.4) for 5 min twice. Cells were then stained with an anti-5mC antibody (Supplementary Table S5) overnight at 4ºC in 1% normal goat serum and 0.3% Triton-X100 in PBS, followed by staining with Alexa Fluor 488-labeled anti-mouse IgG (1:1000) for 1 h at RT in 1% normal goat serum in PBS, and then by staining with an anti-HA antibody for 2 h at RT and Alexa Fluor 647-labeled anti-rabbit IgG (1:1000) for 1 h at RT, both in 1% normal goat serum in PBS. Staining with 4′,6-diamidino-2-phenylindole (DAPI) (1 μg/ml) was performed during the final antibody incubation steps. Hoechst staining (10 μg/ml) was performed after the final antibody incubation step. Fluorescence images were acquired using a FV1000 laser confocal microscope (Olympus) or fluorescence microscope　BZ-X810 (KEYENCE) and quantification analysis was performed using images acquired by FV1000.

Fluorescent intensities of indicated target proteins in nuclear regions were normalized to those of DAPI signal using FV10-ASW software (Olympus). For colocalization analysis between LC3 and NCOA4, and LC3 and Ferritin, fluorescence images were acquired with a 60× objective lens at a size of 1600 × 1600 pixels. Pearson's correlation coefficient (PCC) between pixel intensities in the cytoplasmic region of an individual cell from two fluorescence images was calculated using the Coloc 2 plugin of the Fiji (ImageJ) software.

### RNA–seq

Total RNA was purified from cells using RNeasy Lipid Tissue Mini Kit (QIAGEN, 74804), and library preparation was carried out using KAPA Stranded mRNA–seq Kit (KAPA BIOSYSTEMS, KK8400) according to the manufacturers’ protocols. For each library, DNA concentration was measured by Qubit dsDNA HS Assay Kit (Thermo Fisher, Q32854) and fragment size was measured by High Sensitivity DNA kit for Bioanalyzer (Agilent, 5067-4626) or D1000 ScreenTape assay kit for TapeStation (Agilent, 5067-5582, 5067-5583). Paired-end sequencing was performed on a NextSeq 500 platform (Illumina) for 43 × 2 cycles. Hereafter bioinformatic analysis was performed using indicated software/packages with default settings unless specified otherwise. Reads were aligned to mm9 assembly of the mouse genome (NCBI Build 37, obtained from the UCSC Genome Browser website), and expression values either for each gene or transcript (read counting and transcripts per million [TPM] normalized value) were calculated using the STAR (version 2.5.3a) (46)-RSEM (version 1.3.3) (47) pipeline with the following options: –paired-end, –star, –star-gzipped-read-file, –strandedness reverse, –output-genome-bam, –sort-bam-by-coordinate, –estimate-rspd. DEGs between two conditions (false discovery rate-adjusted *P*-value < 0.05, |logFC| > 1) were detected using TCC-iDEGES-edgeR pipeline on R (https://www.R-project.org/, version 3.6.2) package TCC (version 1.26.0) (48), and pathway analysis was conducted with Ingenuity Pathway Analysis (IPA) (QIAGEN). Heatmaps were generated based on TPM values using the heatmap2 library on R (version 4.0.3) package gplots_3.1.1 as follows: removing genes with CV (coefficient of variation) <0.2, log_2_ transformation, calculating Z-scores, omitting ‘N/A’ data, and hierarchical clustering with Ward's method. For visualization on IGV (Integrative Genomics Viewer, version 2.8.7), BAM files for each replicate were merged with samtools (version 1.9) (49) and then converted to bigWig files using the bamCoverage function of deepTools (normalized as counts per million (CPM)), version 3.5.0) (50).

### Whole-genome bisulfite sequencing (WGBS)

Genomic DNA was purified from cells using DNeasy Blood & Tissue Kit (QIAGEN, 69504). PCR-free WGBS libraries were prepared with the TdT-assisted adenylate connector-mediated ssDNA ligation-mediated Post-Bisulfite Adaptor-Tagging (tPBAT) protocol as described previously (51). One hundred nanograms of genomic DNA spiked with 1 ng of unmethylated lambda DNA was served for the library preparation. Each library was indexed with different sequences, and sequencing was performed on an Illumina HiSeq X Ten system (Macrogen Japan Corp.) with 2 × 150 paired-end chemistry. The same amounts of libraries prepared from three independent biological replicates were mixed in a tube, and one lane of sequencing was assigned per mix. Note that data of three replicates were merged to increase read depth for the downstream analysis. Sequenced reads were mapped on the reference comprised of mouse mm9 and lambda phage with Bmap as described previously (51). The alignments were summarized and exported to bedGraphFiles with in-house developed programs described previously (51). The summary of basic metrics of the DNA methylome data produced in the current study is provided in Supplementary Tables S6 and S7.

### Genome-wide analysis of differentially methylated regions (DMRs) using metilene

The entire WGBS data were analyzed using the metilene software tool (version 0.2-8) to detect DMRs (*q*-value < 0.05) in the whole genome by comparing the 2 conditions (52). Motif analysis for DMRs was carried out using the findMotifsGenome.pl of the HOMER software (version 4.11) (53). Aggregation plot for DNA methylation changes around DMRs was generated as described for Chromatin immunoprecipitation followed by high-throughput sequencing (ChIP-seq) but using the bigWig files converted from bedGraph files by the bedGraphToBigWig program (downloaded from the UCSC Genome Browser website, version 2.8) as well as the bigwigCompare function of deepTools (version 3.5.0). 3D plots for methylation levels of all CpG across three conditions were generated using the Perl and R package ‘rgl’ (function plot3d).

### WGBS data analysis within 1 kb upstream of the transcription start site (TSS)

To evaluate CpG methylation levels within 1 kb upstream from TSSs, the following analysis was conducted using custom Perl scripts (available with test data at https://github.com/j-kohmaru-gunma/NGS_Program/tree/main/Suzuki_et_al_WGBS). First, CpGs located within 1 kb upstream from all TSSs were screened from the original data containing methylation levels and coverage/read depths for all CpGs (in a bedGraph format). After omitting CpGs having <5 or >1000 coverage/read depths, changes in methylation levels on Day 8 were calculated by subtracting from those on Day 0. Finally, genes having three or more CpGs that showed more than a 50% reduction in methylation levels from Day 0 were identified. Expression levels of those genes were visualized using a heatmap as described above for RNA–seq, and pathway analysis was conducted by IPA.

### WGBS data analysis within enhancer regions

Published peak-calling data for H3K27ac ChIP-seq (GSE21365) (54) and DNase I hypersensitive sites (DHSs) (GSE27826) (55) in 3T3-L1 cells were downloaded from Gene Expression Omnibus. For individual datasets, overlapping peaks across the time points were merged using the merge function of bedtools (version 2.28.0; H3K27ac: 71228 peaks; DHS: 46747 peaks), and the regions 500 bp upstream and downstream of each peak center were considered to be putative enhancers. DNA methylation levels in these enhancers were analyzed by applying custom Perl scripts used for the TSS regions. The above merged peak files in a bedGraph format were applied to the script Enhancer_Peak_bed.pl (https://github.com/j-kohmaru-gunma/NGS_Program/tree/main/Suzuki_et_al_WGBS) to set the enhancer endpoint 500 bp downstream of each peak center. The resulting files were used for the same analysis as for the TSS regions described above. Motif analysis was performed as described above with the HOMER software (version 4.11), and all H3K27ac and DHS peaks were used as Custom Background Regions for ChIP-seq and DHSs, respectively. Aggregation plots showing the distribution patterns for DNA methylation around the putative enhancers were generated using deepTools (version 3.5.0), as described above. In addition to analyzing all enhancers throughout the genome, further analysis was performed focusing on enhancers that are activated only after the induction of differentiation. The peaks of H3K27ac and DHS that were detected before differentiation induction (Day 0 and earlier) were omitted using the intersect function of bedtools, and the remaining regions were defined as differentiation-specific enhancers (H3K27ac: 25264 peaks; DHS: 36980 peaks).

### ChIP

ChIP was performed essentially as described (17,25). Cells were fixed with 0.5% formaldehyde at RT for 10 min, followed by quenching with glycine at the final concentration of 0.2 M. Cell pellets were resuspended in Hypotonic Buffer (10 mM HEPES–KOH, pH 7.5, 1.5 mM MgCl_2_, 10 mM KCl, 1 mM EDTA and 1 mM EGTA supplemented with 1 mM PMSF and Protease Inhibitor Cocktail (NACALAI TESQUE, 04080-11)) and then passed through a 22 G needle 10 times. After centrifugation, nuclear pellets were collected and resuspended in 2 ml of 1:4 mixture of SDS Lysis Buffer (50 mM Tris–HCl, pH 8.0, 10 mM EDTA, and 1% SDS) and ChIP Dilution Buffer (16.7 mM Tris–HCl, pH 8.0, 167 mM NaCl, 1.2 mM EDTA, 1.1% Triton X-100, 0.01% SDS) supplemented with the protease inhibitors above. Sonication was carried out to obtain 200–300 bp DNA fragments using a SONIFIER 250 (Branson) with the following setting: output 4, duty cycle 60%, 20 s × 15 times. Sonicated lysates were cleared by centrifugation, and protein concentrations were measured as described above. A total protein of 100 μg for ChIP–qPCR and 500 μg for ChIP–seq of lysate was diluted with SDS Lysis Buffer and ChIP Dilution Buffer with protease inhibitors to be 1:9 ratio. Antibodies (Supplementary Table S5) were pre-bound to 50 μl of Dynabeads Protein G (Thermo Fisher, DB10004) and added to the lysates and rotated at 4ºC for 2 h for ChIP–qPCR or overnight for ChIP–seq. The beads were successively washed twice with the following buffers: Low Salt ChIP Wash Buffer (20 mM Tris–HCl, pH 8.0, 150 mM NaCl, 2 mM EDTA, 1% Triton X-100, and 0.1% SDS), High Salt ChIP Wash Buffer (20 mM Tris–HCl, pH 8.0, 500 mM NaCl, 2 mM EDTA, 1% Triton X-100, and 0.1% SDS), LiCl ChIP Wash Buffer (10 mM Tris–HCl, pH 8.0, 250 mM LiCl, 1 mM EDTA, 1% IGEPAL CA-630 and 1% sodium deoxycholate), and TE Buffer (10 mM Tris–HCl, pH 8.0, and 1 mM EDTA). The beads were then resuspended in 200 μl of buffer containing 1% SDS and 100 mM NaHCO_3_, treated with 200 μg/ml proteinase K at 42ºC for 2 h, and incubated at 65ºC for more than 4 h for reverse-crosslinking. DNA fragments were purified using QIAquick PCR Purification Kit (QIAGEN, 28106), and DNA concentrations were measured using Qubit dsDNA HS Assay Kit. ChIP–qPCR was carried out using the primers in Supplementary Table S8 by the standard curve method. To assess enrichment, %input was calculated relative to the input control.

### ChIP using mouse epididymal white adipose tissue (WAT)

All animal experiments were approved by the Gunma University Ethics Committee for Animal Experiments (protocol approval number: 22–051). Six 8-week-old male C57BL/6N mice (Japan SLC), housed in a humidity- and temperature-controlled specific pathogen-free facility with a 12-h light/dark cycle, were fed *ad libitum* on a high-fat, high-cholesterol diet (Research Diet, D12079B) for 2 weeks, with or without DFO treatment during the same period (3 mice each). DFO treatment was performed by intraperitoneal injection of 100 mg/kg of body weight per day (mg/kgBW/day), and the vehicle group received the same amount of PBS (10 ml/kgBW/day) intraperitoneally. Mice were then sacrificed by cervical dislocation, and epididymal WAT was harvested for ChIP analysis. The resulting epididymal WAT was minced >100 times with scissors, fixed in 0.5% formaldehyde at RT for 10 min, quenched with glycine (final concentration: 0.2 M), washed with ice-cold PBS, and homogenized on ice 50 times in hypotonic buffer using a Wheaton Dounce homogenizer with a loose pestle. After centrifugation at 1000 × *g* for 7 min at 4°C, the pellet was collected and resuspended in 2 ml of SDS Lysis Buffer diluted 5 × with ChIP Dilution Buffer containing protease inhibitors and used for the sonication step described in the ChIP section.

### ChIP–seq

ChIP–seq libraries were prepared using ThruPLEX DNA-Seq Kit (TAKARA BIO, R400674), and 75-cycle single-end sequencing was performed as described above for RNA–seq. Reads were aligned to the mm9 mouse genome using Bowtie 2 (version 2.2.9) (56). Mapped reads having < 40 MAPQ were omitted (samtools view -bhS -F 0 × 4 -q 40 | samtools sort), and duplication removal was then carried out using the markdup -r function of samtools (version 1.11). Analysis of ChIP–seq signals around TSSs was performed on DEGs upregulated from Day 0 to Day 2 (DFO(−)) in RNA–seq as follows. First, ChIP–seq signals around TSSs (±5 kb) of these DEGs were calculated using featureCounts (version v1.5.0-p2) (57) followed by CPM normalization with R (version 4.0.3). Note that some genes have multiple TSSs, either overlapping or discrete. In gene-level analysis, multiple ‘TSSs ±5 kb’ regions, if any, were merged for each gene, and read counting was conducted, followed by normalization with length. For analyzing individual TSSs, reads were counted for each TSSs ±5 kb, even when overlapping with neighboring regions (using -O option of featureCounts). Then, using mean CPM scores from two replicates as an input, fuzzy c-means clustering was performed with the Mfuzz package (version 2.50.0) (58) on R (version 3.6.2), in which the number of clusters was determined as 7 based on the minimum distance Dmin between cluster centroids. For heatmap generation, Z-scores were calculated based on mean CPM values and visualized as described for RNA–seq. GO enrichment analysis on the clusters obtained was performed using clusterProfiler (version 3.18.1) (59) with org.Mm.eg.db_3.12.0 database on R (version 4.0.3) for selected GO terms. For principal component analysis (PCA), bigWig files were generated with the bamCoverage function and subjected to the computeMatrix and plotPCA functions in deepTools (version 3.5.0). To visualize ChIP–seq data on IGV, bigWig files were generated as described for RNA–seq but with the following option in the bamCoverage function: -e 500. To visualize changes in ChIP–seq signals around DMRs, merged BAM files from two replicates were normalized with those of Day 0 samples and converted into bigWig files using the bamCompare function of deepTools (version 3.5.0) with the following options: -e 500 –operation subtract –normalizeUsing CPM –scaleFactorsMethod None. Heatmap and aggregation plot around DMRs (±3 kb from center of DMR) were generated using the computeMatrix (–skipZeros –binSize 10) and plotProfile functions in deepTools. Peak calling for H3K4me3 was carried out using the software MACS2 (version 2.7.1) (60) with the following options: –min-length 400 -q 0.000001 -g mm. Detection of overlapped peaks was done using the findOverlapsOfPeaks of chipPeakAnno (version 3.20.1) (61).

### DNA methylation analysis

Genomic DNA was purified using DNeasy Blood & Tissue Kit at indicated days of differentiation. Bisulfite conversion of genomic DNA was performed with 500 ng of genomic DNA using EpiTect Plus DNA Bisulfite Kit (QIAGEN, 59124) as the manufacture's manual. Target sequences were PCR-amplified using KOD -Multi & Epi- (TOYOBO, KME-101) with specific primer sets targeting the coding strands after the bisulfite conversion, which were designed with MethPrimer (https://www.urogene.org/methprimer/) (62). PCR products were subcloned into pCR-Blunt II TOPO vector (Thermo Fisher, 451245) or pCR4-Blunt TOPO vector (Thermo Fisher, 450031). Plasmids were isolated by boiling transformed E. coli for 1 min in buffer containing 0.7 mg/ml lysozyme, 10 mM Tris–HCl, pH 7.5, 63 mM EDTA, 2.5 M LiCl, and 4% Triton X-100 with immediate cooling on ice and purified by isopropanol precipitation. The inserts were PCR-amplified by M13 forward and reverse primers using KOD-Plus Neo (TOYOBO, KOD-401) and subjected to Sanger sequencing. All primers for DNA methylation analysis are listed up in Supplementary Table S9. Sequence data was analyzed using QUantification tool for Methylation Analysis (QUMA) (http://quma.cdb.riken.jp/) (63).

### JMJD1A activity assay using homogeneous time-resolved fluorescence (HTRF)

HTRF, a fluorescence resonance energy transfer (FRET)-based technology, was performed using a biotinylated dimethyl-histone H3K9 (1–21) peptide substrate (Anaspec #63678) or a biotinylated monomethyl-histone H3K9 (1–21) peptide substrate (Anaspec #65349) and a europium cryptate-labeled anti-unmethylated histone H3K9-specific monoclonal antibody (PerkinElmer, anti-H3K9me0-Eu[K] #61KB0KAE). The recombinant JMJD1A (13.5 nM) (BPS Bioscience #50130, lot #120511-G2) and the substrate (100 nM) were reacted in enzymatic buffer (50 mM HEPES–NaOH, pH 7.0, 0.01% Tween 20, serial concentrations [0, 3.13, 12.5, and 50.0 μM] of ammonium iron (II) sulfate, 500 μM ascorbic acid, 500 μM α-ketoglutarate [α-KG], and 1 × Halt protease inhibitor cocktail [Thermo Fisher]) at RT for 1 h. For terminating and measuring JMJD1A catalyzed demethylation, the detection reactions were supplemented with both anti-H3K9me0-Eu(K) and XL665-conjugated streptavidin (SA-XL665) dissolved in EDTA containing detection buffer (PerkinElmer, #62SDBRDD). Each reaction was incubated for 1 h at RT before measuring the time resolved-FRET signal at 620 nm and 665 nm using an ARVO X5 microplate reader (PerkinElmer).

The following equation was used to calculate Delta F % (DF%): DF% = ([665 nm/620 nm of Enzyme (+) condition]/[665 nm/620 nm of Enzyme (−) condition]−1) × 100

### *In vitro* H3K9me2 demethylation assay using purified cell-derived JMJD1A

To generate 3T3-L1 cells stably expressing Flag-Twin-Strep-tagged H3K9me2 demethylase, an artificially synthesized Flag-Twin-Strep sequence (IDT, gBlock) was incorporated into the shRNA-resistant retrovirus plasmid by Gibson assembly (Supplementary Table S2), and the plasmids were transfected into Platinum-E packaging cells to produce retroviruses as described above. Cells were then infected with the produced retrovirus, and infected cells were selected by adding 10 μg/ml blasticidin S. Cells expressing Flag-Twin-Strep-tagged demethylase were collected in a precipitation buffer containing 50 mM HEPES–KOH, pH 7.9, 150 mM NaCl, 1.5 mM MgCl_2_ and 1% Nonidet-P40 supplemented with EDTA-free protease inhibitor cocktail (Roche, 05056489001), and then sonicated using a Branson Sonifier SFX150 (Emerson) with the following setting: 4 cycles of 55% continuous amplitude for 10 s with a 50 sec interval. Flag-Twin-Strep-tagged demethylases were precipitated with prewashed StrepTactin beads (IBA Lifesciences, 2-5010-002) for 2 h at 4ºC, and then washed with the precipitation buffer and PBS. A total of 1 μg synthesized H3K9me2 peptide (Epigentek, R-1026) was added to the tube containing the Flag-Twin-Strep-tagged demethylases binding to StrepTactin beads in a reaction buffer (50 mM HEPES–KOH, pH 7.5, 1 mM α-KG and 2 mM ascorbic acid) in the presence or absence of 70 μM ferrous ammonium sulfate, and incubated at 37ºC for 1 h (preadipocytes) or 10 min (Day 2 samples). Samples were boiled at 95ºC for 5 min in 1 × Laemmli SDS sample buffer containing 25 mM DTT and protease inhibitor cocktail (Roche, 05056489001), and were then applied for immunoblotting as above.

### Measurement of α-KG

Nuclear α-KG concentration during differentiation was measured as described previously (24). For liquid chromatography-tandem mass spectrometry (LC–MS/MS) analysis, metabolites were extracted from cells with the solvent of water: methanol: chloroform (1:2.5:1, v/v/v) containing 10 μM 2-(*N*-morpholino) ethanesulfonic acid (2-MES) as an internal standard. The extracts were purified by using a CaptivaND Lipid filter plate (Agilent) according to the manufacturer's instruction. The filtrates were dried up by a vacuum evaporator, re-suspended with distilled water, and used for the detection of metabolites by LC–MS/MS. LC–MS/MS analysis of α-KG was performed by using a triple quadrupole mass spectrometer coupled with a liquid chromatograph (LCMS-8050 system, Shimadzu). The cell extract was separated on a MastroSP column (2.1 mm × 100 mm, 5 μm, Shimadzu) by using a gradient of solvent A (10 mM ammonium acetate in water/acetonitrile 90/10) and solvent B (50 mM ammonium acetate in water/acetonitrile 80/20) with a flow rate of 0.5 ml/min. The initial solvent composition was 0% solvent B, and the following solvent gradient was applied: 0% solvent B for 4 min, increased linearly to 60% solvent B from 4 to 9 min, to 100% solvent B from 9 to 11 min, held at 100% solvent B for 2 min, then returned to 0% solvent B and held for 5 min. The column was maintained at 40°C. The separated analytes were ionized by electrospray ionization, and then measured by the mass spectrometer with the selected reaction monitoring (SRM) mode. The SRM transitions were *m*/*z* 144.9 > 101.1 [M−H]^−^ for α-KG and *m*/*z* 194.0 > 80.2 [M−H]^−^ for 2-MES (internal standard). The peak height of α-KG was divided by that of the internal standard and is presented as the relative ion intensity.

### Statistical analysis

Statistical analysis was performed using R software (version 4.1.1). Statistical difference between two groups were examined by the two-tailed Student's *t*-test. For multiple comparison, groups were compared by one-way analysis of variance (ANOVA), followed by the post-hoc Tukey–Kramer test for comparison among groups or the post-hoc Dunnett test for comparison of groups with a specific control. Alternatively, the Kruskal–Wallis test was applied, followed by the post-hoc Steel–Dwass test. Data are expressed as mean ± standard error of the mean (s.e.m.) or as median with individual plots. *P* < 0.05 was considered statistically significant.

## RESULTS

### Requirement of iron in the early stage of adipocyte differentiation

**Figure 1.**
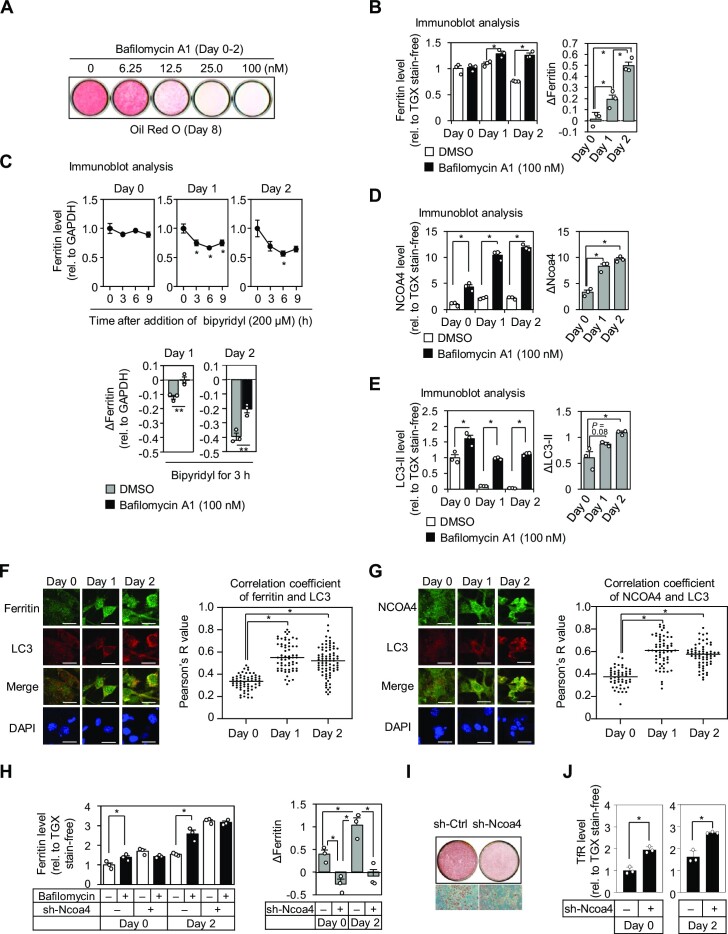
Ferritinophagy is induced during the early stage of adipocyte differentiation. (**A**) Adipocyte differentiation of 3T3-L1 cells was induced with the addition of bafilomycin A1 at the indicated concentrations for the first 2 days, and ORO staining was performed on Day 8. (**B**) The lysosomal flux of ferritin was measured by immunoblot analysis on Days 0, 1 and 2 using whole cell lysates from 3T3-L1 cells. Cells were treated with DMSO or 100 nM bafilomycin A1 for 24 h prior to the collection of whole cell lysates (*n* = 3 biological replicates). Ferritin levels were normalized to the total protein level quantified by TGX stain-free gel (left). Ferritin flux to the lysosomes was calculated by subtracting the ferritin level in DMSO-treated cells from that in bafilomycin A1-treated cells (right). Data are shown as the mean ± s.e.m. The two-tailed Student's *t*-test (left) or one-way ANOVA followed by the Tukey-Kramer test (right) was performed for statistical analysis. *P < 0.05. The immunoblot images are shown in Supplementary Figures S1B and S10. (**C**) On the indicated day of differentiation, 3T3-L1 cells were treated with 200 μM 2,2-bipyridyl for indicated hours (top) or 200 μM 2,2-bipyridyl and either 100 nM bafilomycin A1 or DMSO for 3 h (bottom). Ferritin levels in whole cell lysates were quantified by immunoblot analysis, and normalized to the GAPDH level (*n* = 3 biological replicates) (top). The change in the ferritin level in response to 2,2-bipyridyl treatment was calculated by subtracting the level before treatment from the level 3 h after treatment (*n* = 3 biological replicates) (bottom). Data are shown as the mean ± s.e.m. One-way ANOVA followed by the Tukey–Kramer test (top) or the two-tailed Student's *t*-test (bottom) was performed for statistical analysis. *P < 0.05, **P < 0.01. The immunoblot images are shown in Supplementary Figures S1C, D, and S10. (D, E) The lysosomal flux of NCOA4 (**D**) and LC3-II (**E**) was measured by immunoblot analysis as performed in (B) (*n* = 3 biological replicates). Data are shown as the mean ± s.e.m. The two-tailed Student's *t*-test (D, left, E, left) or one-way ANOVA followed by the Tukey-Kramer test (D, right, E, right) was performed for statistical analysis. *P < 0.05. The immunoblot images are shown in Supplementary Figures S1C, D, and S10. (F, G) On Days 0, 1 and 2, 3T3-L1 cells were treated with 100 nM bafilomycin A1 for 6 h, and immunostained for LC3 and ferritin (**F**), or LC3 and NCOA4 (**G**). Scale bar, 10 μm. For evaluating the colocalization of LC3 and ferritin (F) or NCOA4 (G), Pearson's *R*-values in the cytoplasmic region of each cell were calculated in 50 to 80 cells (F, right, G, right). The horizontal line indicates the median value. The Kruskal–Wallis test followed by the Steel–Dwass test was performed for statistical analysis. *P < 0.05. The immunoblot images are shown in Supplementary Figures S1B and S10. (H, I) The NCOA4-KD cell line established by stably expressing an shRNA against Ncoa4 mRNA (sh-Ncoa4), and its control cell line (sh-Ctrl) were induced to undergo adipocyte differentiation. The lysosomal flux of ferritin was measured on Days 0 and 2 by immunoblot analysis, as performed in (B) (*n* = 3 biological replicates) (**H**). ORO staining was performed on Day 8 (**I**). Data are shown as the mean ± s.e.m. The two-tailed Student's *t*-test (H, left) or one-way ANOVA followed by the Tukey–Kramer test (H, right) were performed for statistical analysis. *P < 0.05. The immunoblot images of (H) are shown in Supplementary Figures S1F and S10. (**J**) Whole cell lysates of the NCOA4-KD cell line (sh-Ncoa4) and the control cell line were subjected to immunoblot analysis using an anti-transferrin receptor (TfR) antibody on Days 0 and 2 (*n* = 3 biological replicates). TfR levels were normalized to the total protein level quantified by TGX stain-free gel. Data are shown as the mean ± s.e.m. One-way ANOVA followed by the Tukey–Kramer test was performed for statistical analysis. *P < 0.05. The immunoblot images are shown in Supplementary Figure S10.

**Figure 2.**
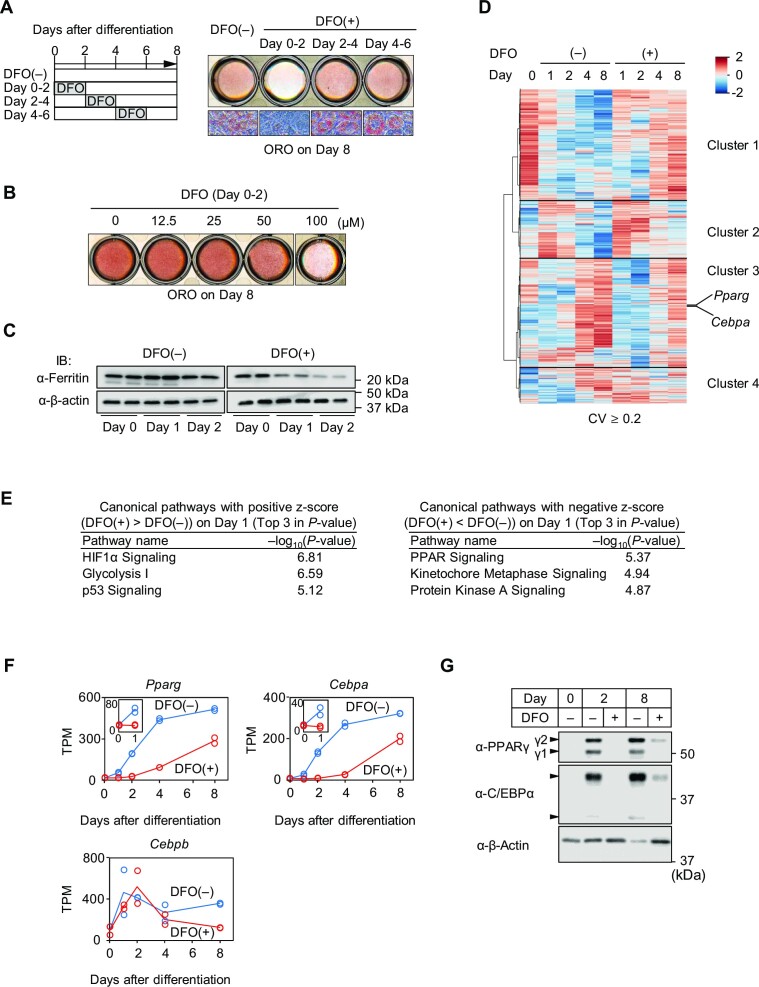
Transient iron depletion during the early stage of adipocyte differentiation suppresses terminal differentiation of 3T3-L1 cells. (**A**) Cells were treated with vehicle (−) or DFO (100 μM) for the indicated times during adipocyte differentiation as schematically illustrated (left). Cells were stained with ORO on Day 8, and a scanned picture of the cell culture plate (top right) and bright field microscopy images (bottom right) are shown. (**B**) Cells were treated with the indicated concentrations of DFO for the first 2 days of adipocyte differentiation, and stained with ORO on Day 8. (**C**) Cells were differentiated into adipocytes with (+) or without (−) 100 μM DFO. Protein levels of ferritin and β-actin at the indicated time points were determined by immunoblot analysis using whole cell extracts. Uncropped images are shown in Supplementary Figure S10. (**D**) RNA-seq heatmap depicting the comparison of gene expression patterns at each time point (Days 0, 1, 2, 4 and 8) for cells treated with vehicle (−) or DFO (100 μM) for the first two days during adipocyte differentiation. For each gene, z-scores were calculated based on log2-transformed mean TPM values, and are shown along the color scale. Expression patterns of the genes, except for genes with a coefficient of variation (CV) of <0.2, were classified by hierarchical clustering. A total of 4907 expressed genes were classified into four groups (cluster 1: 1742 genes; cluster 2: 901 genes; cluster 3: 1692 genes; cluster 4: 572 genes). Both *Pparg* and *Cebpa* were in cluster 3 as indicated. (**E**) The top 3 canonical pathways ranked by *P*-value for DEGs that were upregulated (positive *z*-score) or downregulated (negative *z*-score) by DFO treatment on Day 1. (**F**) Transcriptional changes of adipogenic regulatory genes (*Pparg*, *Cebpa*, and *Cebpb*) determined by RNA-seq. Data are presented in TPM and circles indicate two biological replicates. Insets show the changes from Day 0 to Day 1. (**G**) Protein levels of PPARγ, C/EBPα and β-actin at the indicated time points during 3T3-L1 cell differentiation were determined by immunoblot analysis using whole cell extracts. Uncropped images are shown in Supplementary Figure S10.

Several lines of evidence support the idea that both autophagy and iron are important for adipocyte differentiation (1,21). However, the role of autophagic degradation of the iron-storage protein ferritin (ferritinophagy) in adipocyte differentiation remained unclear. Therefore, we investigated the significance of ferritinophagy in adipocyte differentiation. First, we blocked the lysosomal degradation of ferritin by adding the lysosomal inhibitor bafilomycin A1 (45,64). Interestingly, the terminal differentiation of adipocytes was significantly inhibited by bafilomycin A1 in a dose-dependent manner, as determined by ORO staining on day 8 of differentiation (Day 8) (Figure 1A). Of note, this inhibition occurred when bafilomycin A1 was added for the first 2 days in the entire process of differentiation for 8 days. Similar inhibition of adipogenesis was observed by the treatment of PIK-III, a specific inhibitor of vacuolar protein sorting 34, which plays a central role in the initiation of autophagy (11) (Supplementary Figure S1A). These data are consistent with previous reports that autophagy is crucial during the early stage of 3T3-L1 preadipocyte differentiation (18,19) and suggested the importance of lysosomal ferritin degradation in the early stage of adipocyte differentiation. Therefore, we next analyzed the lysosomal flux of ferritin in the early stage of adipocyte differentiation, and found that the lysosomal flux of ferritin is gradually increased after the induction of differentiation (Figure 1B, right; Supplementary Figure S1B). Accelerated ferritin degradation was also clearly observed when the translation of ferritin was inhibited by adding 2,2'-bipyridyl (65) (Figure 1C, top; Supplementary Figure S1C). In addition, this degradation was inhibited by bafilomycin A1, indicating that ferritin degradation is mediated by lysosomal degradation (Figure 1C, bottom; Supplementary Figure S1D). To verify that ferritin degradation during the early stage of adipocyte differentiation is mediated by ferritinophagy, we analyzed the lysosomal flux of NCOA4, a specific cargo receptor of ferritinophagy, together with the flux of LC3, a marker of isolation membranes and autophagosomes (6,9,11). Importantly, the lysosomal flux of both NCOA4 and LC3 was increased during the first 2 days of differentiation (Figure 1D, E; Supplementary Figure S1B). In addition, this was associated with increased colocalization of ferritin and NCOA4 with LC3 after the induction of adipocyte differentiation (Figure 1F, G). Thus, ferritinophagy is induced during the early stage of adipocyte differentiation. We next generated NCOA4 knockdown (NCOA4-KD) cells by stably expressing shRNA in 3T3-L1 preadipocytes (Supplementary Figure S1E). Ferritin degradation was significantly suppressed in these cells (Figure 1H; Supplementary Figure S1F). However, the adipocyte differentiation of NCOA4-KD cells was mildly but not completely suppressed (Figure 1I). We postulated that this is presumably due to a compensatory increase in iron uptake in cells with stable knockdown of NCOA4. This was supported by the finding that basal protein levels of the transferrin receptor were higher in NCOA4-KD cells than in control cells (Figure 1J). Taken together, these results indicate that iron is highly required during the early stage of adipocyte differentiation, which is accompanied by a corresponding increase in ferritinophagy and a concomitant increase in extracellular iron uptake. Therefore, we next treated cells with the iron chelator DFO to analyze the combined effects of both the depletion of stored iron and reduced iron uptake. When 3T3-L1 cells were treated with DFO during the entire course of differentiation, their terminal differentiation was markedly suppressed (Supplementary Figure S2A), as reported previously (3–5). However, no apparent suppression was observed when DFO treatment was started on Day 2 (Supplementary Figure S2A). Further analysis demonstrated that DFO treatment during the first 2 days of differentiation was necessary and sufficient to suppress adipocyte differentiation (Figure 2A; Supplementary Figure S2B), and its effect was dose-dependent (Figure 2B). This is in important agreement with the finding that ferritinophagy is crucial for the early stage of adipocyte differentiation. Indeed, ferritin levels were gradually decreased over the first 2 days of differentiation in response to DFO treatment (Figure 2C; Supplementary Figure S2C). Thus, ferritin acts as an intracellular iron sink under the conditions of increased iron demand during the early stage of adipocyte differentiation. Therefore, in our subsequent experiments, we analyzed the effects of DFO treatment during the first 2 days of adipocyte differentiation.

### DFO treatment regulates the mRNA expression of regulatory genes of adipocyte differentiation

We next determined the effects of iron depletion on gene expression profiles. The adipocyte differentiation of 3T3-L1 cells was induced either with or without the addition of DFO for the first 2 days, and mRNA was extracted from the cells on Days 0, 1, 2, 4 and 8. RNA-seq analysis detected 4907 variably expressed genes classified into four groups (Figure 2D). Interestingly, the number of genes upregulated by DFO (cluster 1) was comparable to the number of genes downregulated by DFO (cluster 3). The top three pathways activated by DFO on Day 1 included the previously reported iron-induced pathways (6,66,67), such as the hypoxia-inducible factor 1α (HIF-1α) signaling, the glycolytic pathway, and the p53 signaling (Figure 2E, left). In contrast, pathways suppressed by DFO included the PPAR signaling pathway, kinetochore metaphase signaling, and protein kinase A signaling (Figure 2E, right). Importantly, the PPAR signaling pathway is crucial for adipocyte differentiation, as PPARγ is the master regulator of adipocyte differentiation (13). Consistently, protein levels of PPARγ and mRNA expression of its encoding gene *Pparg* were severely repressed by DFO (Figure 2F, G). This was associated with reduced mRNA expression of *Cebpa* and protein expression of C/EBPα, as PPARγ and C/EBPα regulate each other's transcription in a positive-feedback manner during adipocyte differentiation (Figure 2F, G). In contrast, expression of the *Cebpb* transcript, which is induced at the very early phase of differentiation (13), did not show any significant reduction (Figure 2F).

### Iron-dependent demethylation of repressive histone marks during adipocyte differentiation

**Figure 3.**
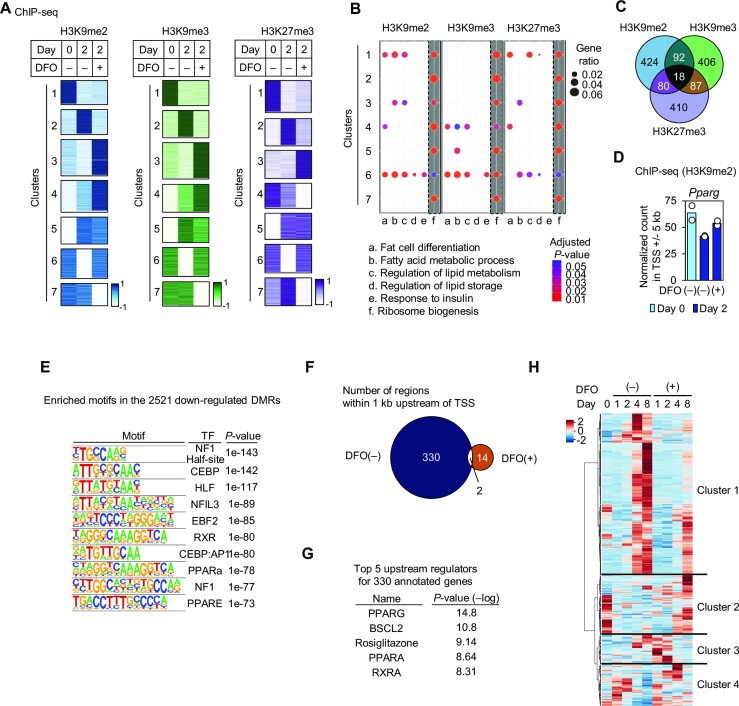
Iron-dependent demethylation of histones and DNA correlates with the expression of adipocyte differentiation-associated genes. ChIP-seq analysis against repressive histone marks (H3K9me2, H3K9me3, and H3K27me3) was performed. 3T3-L1 cells were induced to differentiate with [DFO(+)] or without [DFO(−)] the addition of 100 μM DFO during the first two days of differentiation, and samples were collected at the indicated time points. (**A**) The ChIP-seq signals of H3K9me2, H3K9me3, and H3K27me3 were calculated as the normalized count per million (CPM) in the TSS ±5 kb region of each DEG with upregulated expression from Day 0 to Day 2 in the DFO(−) condition. The patterns of histone modifications among the three groups, i.e. Day 0, DFO(−) on Day 2, and DFO(+) on Day 2, were classified into seven clusters based on the z-score calculated from the mean CPM of duplicate cultures. (**B**) GO enrichment analysis was performed across the 7 clusters in (A) for the terms selected as relevant to adipocyte biology using the ubiquitously distributed ‘ribosome biogenesis’ (highlighted in gray) as a control. For reference, both a color intensity scale of the adjusted *P*-value and a size scale of the gene ratio are included. (**C**) Venn diagram depicting the number of overlapping genes in cluster 6 of H3K9me2, H3K9me3, and H3K27me3 in (A). (**D**) The normalized CPM of H3K9me2 ChIP-seq signals in the TSS ± 5 kb of *Pparg*. The circles represent two individual replicates for each treatment. (**E**) WGBS was performed in triplicate using 3T3-L1 cells differentiated into adipocytes with or without 100 μM DFO treatment for the first two days of differentiation. Metilene software detected 2521 DMRs as significantly decreased on Day 8. The enriched motifs among the DMRs are shown. (**F**) Using the WGBS data in (E), the DNA methylation level of the region within 1 kb upstream from the TSS of each gene was analyzed. CpGs that differ in methylation level by more than 50% on Day 8 [DFO(−) or DFO(+)] compared with Day 0 were identified, and the number of regions with 3 or more such CpGs is shown in the Venn diagram. (**G**) Top 5 upstream regulators of 330 iron-dependent demethylated genes in (F). (**H**) The 330 iron-dependent demethylated regions during adipocyte differentiation in (F) were annotated into their flanking genes. RNA-seq heatmap depicting their expression profiles throughout adipocyte differentiation classified into four clusters by Ward's hierarchical clustering method.

Sequential gene expression during adipocyte differentiation is regulated by both epigenetic regulation as well as transcriptional factors, including PPARγ and C/EBPα. As iron is an essential cofactor for demethylases of histones and DNA, we next analyzed the epigenetic modifications altered by DFO in the early stage of adipocyte differentiation. First, whole-cell levels of histone modifications were determined by immunocytochemistry. Repressive histone marks, such as histone H3 lysine 9 di- and tri-methylation (H3K9me2/me3), and H3K27me3 showed a decreasing trend during differentiation (from Day 0 to Day 2), which was inhibited by DFO treatment (Supplementary Figure S3A). In contrast, the levels of H3K27 acetylation (H3K27ac) showed a reciprocal pattern (Supplementary Figure S3A). Next, changes in histone modifications were further analyzed in a genomic region-specific manner. ChIP-seq was performed using either anti-H3K4me3, anti-H3K9me2, anti-H3K9me3, or anti-H3K27me3 antibodies in duplicate samples. PCA of each ChIP-seq data showed a clear separation between the Day 0, Day 2 DFO(−) and Day 2 DFO(+) groups (Supplementary Figure S3B). The obtained ChIP-seq signals were calculated as normalized counts per million in the TSS ±5 kb region of each upwardly expressed gene during the first 2 days of differentiation (Figure 3A; Supplementary Figure S3B). The patterns of changes in histone modifications were classified into 7 clusters (Figure 3A) except for H3K4me3, which showed only limited changes, with its distribution overlapping well among the groups, namely Day 0, Day 2 DFO(−), and Day 2 DFO(+) (Supplementary Figure S3C). Gene ontology (GO) enrichment analysis of terms associated with adipocyte biology across clusters showed that GO terms including ‘fat cell differentiation’ were highly enriched in cluster 6 of H3K9me2 and H3K9me3, and some were also enriched in cluster 6 of H3K27me3, whereas ‘ribosome biogenesis’ was ubiquitously distributed (Figure 3B). Of note, cluster 6 of each histone mark represents a region that becomes demethylated during the first 2 days of differentiation, but not when treated with DFO (Figure 3A). This indicates that the demethylation of repressive histone marks in these regions is iron-dependent, and therefore, we postulated that this is due to requirement of the iron-dependent activation of demethylases. The comparison of genes annotated in each cluster 6 of the repressive histone marks showed that only approximately 15% of the regions overlapped between any pair of histone marks (Figure 3C), indicating that most of the target genes of each histone modification are specific. The above findings were corroborated by individual examples of adipocyte differentiation-associated genes, such as *Pparg* and *Retn* (68) in the clusters regulated by H3K9me2, *Rarres2* (69) and *Mrap* (70) in the clusters regulated by H3K9me3, and *Cebpa* and *G0s2* (71) in the clusters regulated by H3K27me3 (Figure 3D; Supplementary Figure S3D, E).

### Iron-dependent DNA demethylation during adipocyte differentiation

DNA methylation is another epigenetic mark implicated in the regulation of adipocyte differentiation (15). To determine whether the genomic distribution of DNA methylation changes in an iron-dependent manner, we performed WGBS (Supplementary Figure S3F, Supplementary Tables S6 and S7). Overall trends in the genome-wide distribution of DNA methylation were similar between before and after adipocyte differentiation, and between with and without DFO treatment (Supplementary Figure S3F). However, focusing on methylated CpGs, a decreasing trend was observed with adipocyte differentiation, whereas no such decrease was observed upon DFO treatment (Supplementary Figure S3G). This suggested that DNA methylation is altered in a limited number of specific genomic regions. To identify these DMRs within whole-genome sequences during adipocyte differentiation, the metilene software tool (52) was used. When adipocytes were differentiated under normal conditions, i.e. DFO(−), 2521 downregulated DMRs were detected, whereas only 9 downregulated DMRs were detected when adipocytes were differentiated with DFO for the first 2 days, i.e. DFO(+) (Supplementary Figure S3H). The upregulated DMRs were less than 26 in conditions of both with and without DFO treatment (Supplementary Figure S3H). Thus, DNA was iron-dependently demethylated during adipocyte differentiation. Motif analysis of the downregulated DMRs demonstrated highly enriched binding motifs of transcriptional regulators of adipocyte differentiation, including C/EBPs, PPARγ, nuclear factor I (NF1), and early B cell factor 2 (EBF2) (13,16,72) (Figure 3E). To analyze the association between DNA methylation and neighboring gene expression, DNA methylation levels were analyzed in a region 1 kb upstream from the TSS of each gene. A total of 330 demethylated regions were detected after differentiating adipocytes under the normal condition, i.e. DFO(−) (Figure 3F). Pathway analysis of upstream regulators showed high enrichment of PPARγ and its heterodimeric partner, retinoid X receptor, and its ligand rosiglitazone (Figure 3G). When each region was annotated to flanking genes, and their expression profiles were classified into four groups (Figure 3H), more than half of the 330 genes (171 genes) were classified into cluster 1, a group of genes upregulated during differentiation, with lower expression under the DFO(+) condition. Collectively, our results demonstrated that the expression of several genes during adipocyte differentiation is regulated by iron-dependent DNA demethylation.

### JMJD1A mediates iron-dependent demethylation of H3K9me2 in the *Pparg* region

**Figure 4.**
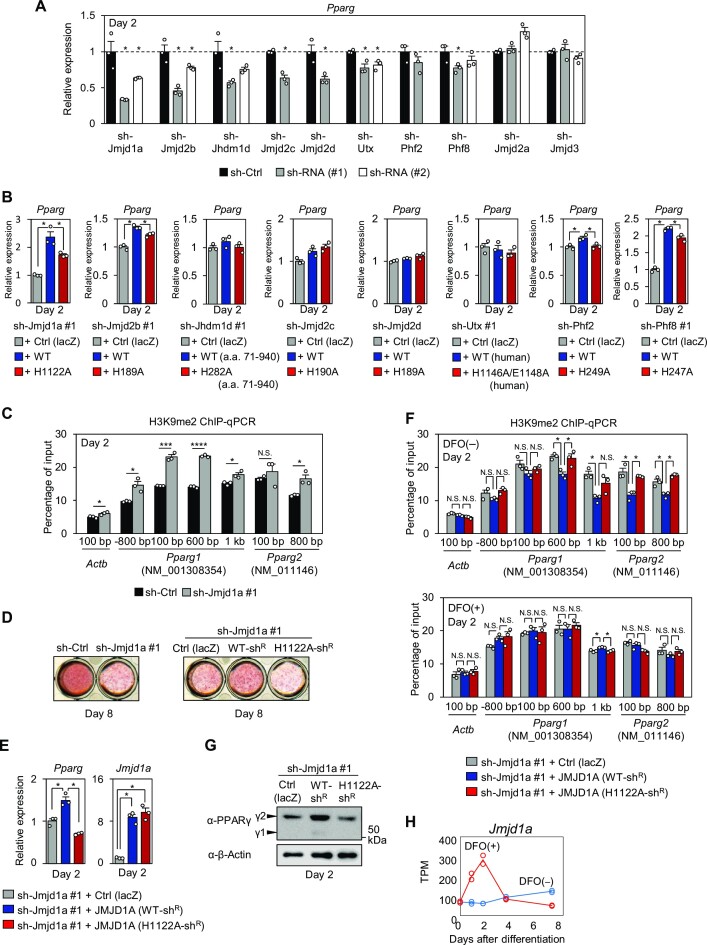
JMJD1A mediates iron-dependent demethylation of H3K9me2 in the *Pparg* region. (**A**) *Pparg* mRNA levels on Day 2 in a series of 3T3-L1 cell lines stably expressing either sh-Jmjd1a, sh-Jmjd2b, sh-Jhdm1d, sh-Jmjd2c, sh-Jmjd2d, sh-Utx, sh-Phf2, sh-Jmjd2a, or sh-Jmjd3 were determined by qPCR (*n* = 3 biological replicates). The mRNA levels normalized to the *Cyclophilin B* level in the cell lines stably expressing an shRNA for each target enzyme (shRNA #1 or shRNA #2) is shown as a ratio to the level of the corresponding control cell line (sh-Ctrl). Data are shown as the mean ± s.e.m. The two-tailed Student's *t*-test was performed for the cell lines expressing either sh-Jmjd2c, sh-Jmjd2d, or sh-Phf2. One-way ANOVA followed by the Dunnett test was performed for the other cell lines. *P < 0.05. (**B**) Each knockdown line was retrovirally transduced with the corresponding enzyme with or without the indicated mutation in the iron-binding site. *Pparg* mRNA levels on Day 2 were measured by qPCR (*n* = 3 biological replicates). The full-length mouse sequence of the corresponding enzyme gene was used for overexpression, except for the partial sequence encoding amino acids (a.a.) 71 to 940 of JHDM1D and the human version for UTX. Data are shown as the mean ± s.e.m. One-way ANOVA followed by the Tukey-Kramer test was performed for statistical analysis. *P < 0.05. (**C**) ChIP-qPCR analysis of H3K9me2 on *Pparg* and *Actb* genes in JMJD1A-KD cells (sh-Jmjd1a #1) and its control cell line (sh-Ctrl) on Day 2 (mean ± s.e.m. of three biological replicates). ChIP signals were presented as a percentage of input DNA. The two-tailed Student's *t*-test was performed for statistical analysis. *P < 0.05, ***P < 0.001, ****P < 0.0001, N.S., not significant. (**D**) ORO staining was performed on Day 8 in JMJD1A-KD cells (sh-Jmjd1a #1), or the JMJD1A-KD cells (sh-Jmjd1a #1) that stably express mouse JMJD1A harboring an shRNA-resistant mutation with [JMJD1A (H1122A-sh^R^)] or without [JMJD1A (WT-sh^R^)] additional mutations in the iron-binding site. (**E**) JMJD1A-KD cells (sh-Jmjd1a #1) overexpressing either lacZ [Ctrl (lacZ)], JMJD1A (WT-sh^R^), or JMJD1A (H1122A-sh^R^) were induced to differentiate, and mRNA levels of *Jmjd1a* and *Pparg* on Day 2 were measured by qPCR (*n* = 3 biological replicates). Data are shown as the mean ± s.e.m. One-way ANOVA followed by the Tukey-Kramer test was performed for statistical analysis. *P < 0.05. (**F**) ChIP-qPCR analysis of H3K9me2 on *Pparg* and *Actb* genes. JMJD1A-KD cells (sh-Jmjd1a #1) overexpressing either lacZ [Ctrl (lacZ)], JMJD1A (WT-sh^R^), or JMJD1A (H1122A-sh^R^) were differentiated with 100 μM DFO [DFO(+)] (bottom) or vehicle [DFO(−)] (top). ChIP-qPCR was performed on Day 2 (mean ± s.e.m. of three biological replicates). One-way ANOVA followed by the Tukey-Kramer test was performed for statistical analysis. *P < 0.05; N.S.: not significant. (**G**) JMJD1A-KD cells (sh-Jmjd1a #1) overexpressing either lacZ [Ctrl (lacZ)], JMJD1A (WT-sh^R^), or JMJD1A (H1122A-sh^R^) were induced to differentiate, and protein levels of PPARγ and β-actin on Day 2 of differentiation were determined by immunoblot analysis using whole cell extracts. Uncropped images are shown in Supplementary Figure S10. (**H**) Adipocyte differentiation of 3T3-L1 cells was induced with or without treatment of 100 μM DFO for the first 2 days, and mRNA levels of *Jmjd1a* during adipocyte differentiation were analyzed by RNA-seq, as described in Figure 2D. Data are presented in TPM and circles indicate two biological replicates.

As inhibition of adipocyte differentiation by DFO treatment was mediated by repressive histone marks, we next sought to identify the histone demethylases responsible for iron-dependent adipocyte differentiation. As the first screening, we knocked down a series of previously reported demethylases of H3K9 or H3K27, and screened them using *Pparg* expression as an indicator. Knockdown of each enzyme except for JMJD2A and JMJD3 resulted in a 15–65% reduction in *Pparg* expression on Day 2 (Figure 4A; Supplementary Figure S4A), which was partially consistent with previous findings (27,35,73–75). Rescue experiments were then performed by overexpressing each enzyme with or without mutations in the iron-binding site (IBD). Overexpression of JMJD1A, JMJD2B, JHDM1D, PHF2, and PHF8 increased *Pparg* expression in their corresponding knockdown cells, whereas their IBD mutants showed reduced effects (Figure 4B; Supplementary Figure S4B). Thus, these enzymes regulate adipocyte differentiation in an iron-dependent manner. JMJD1A showed the most substantial effect among these enzymes and was further investigated. As JMJD1A is a demethylase targeting mono- or di-methylated H3K9, H3K9me2 levels were determined by performing ChIP-qPCR analysis using a JMJD1A-KD cell line (Figure 4C). H3K9me2 levels in the genomic region of *Pparg* were higher in JMJD1A-KD cells than in control cells on Day 2, which was inversely correlated with *Pparg* expression on Day 2 (Figure 4A), and lipid accumulation on Day 8 (Figure 4D, left). To analyze the iron-dependent regulation of H3K9me2 by JMJD1A, we next overexpressed JMJD1A, which is the shRNA-resistant form of JMJD1A harboring a silent mutation, in JMJD1A-KD cells (Figure 4E, right). JMJD1A with a wild-type IBD (WT-JMJD1A) reduced H3K9me2 levels (Figure 4F, top), but JMJD1A containing an IBD with the H1122A mutation (Mut-JMJD1A), which prevents iron binding, did not. This was inversely correlated with the expression levels of *Pparg* mRNA (Figure 4E, left) and PPARγ protein (Figure 4G) on Day 2, and lipid accumulation on Day 8 (Figure 4D, right). Importantly, when treated with DFO, the reduction in H3K9me2 in WT-JMJD1A-expressing cells was reversed to levels comparable to Mut-JMJD1A-expressing cells (Figure 4F, bottom). This reversal in H3K9me2 levels is thought to be due to the modulation of JMJD1A enzyme activity, considering that DFO treatment did not suppress *Jmjd1a* expression in the early stage of adipocyte differentiation (Figure 4H).

### Iron regulates the activity of histone demethylase JMJD1A

**Figure 5.**
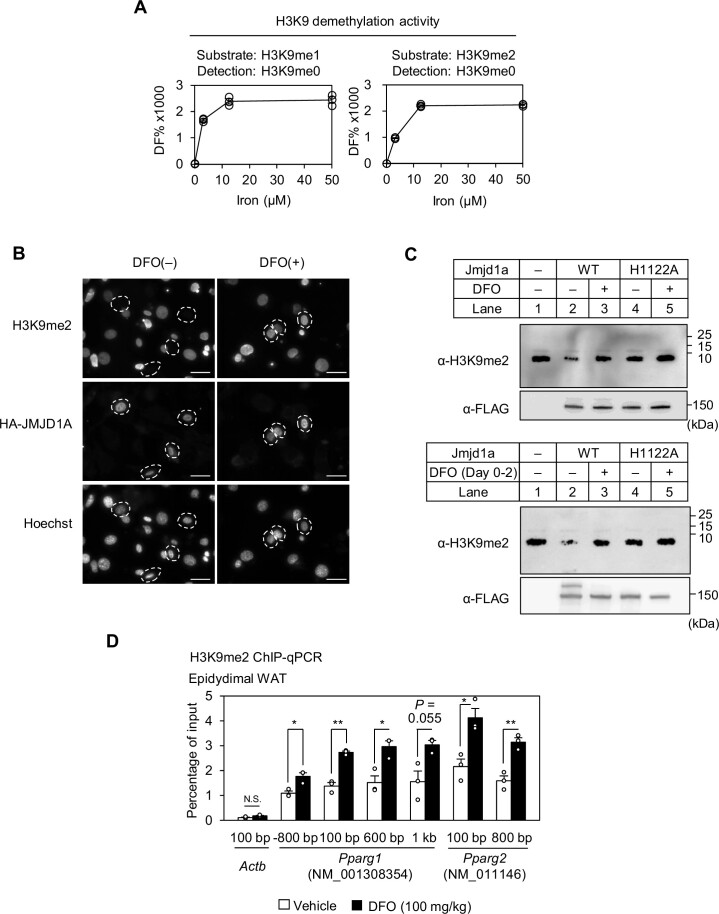
Iron regulates the H3K9me2 demethylase activity of JMJD1A. (**A**) Histone demethylase activity of JMJD1A was determined by the HTRF demethylation assay using the recombinant JMJD1A protein and its substrate (biotinylated-H3K9me1 or biotinylated-H3K9me2). The vertical axis represents DF%: DF% = ([665 nm/620 nm of Enzyme (+) condition] / [665 nm/620 nm of Enzyme (−) condition] −1) ×100). Data are shown as the mean ± SD of 3 technical replicates. (**B**) Cells transfected with pCAG-HA-Jmjd1a were cultured in the presence or absence of 100 μM DFO for 2 days, and immunostained with anti-H3K9me2 and anti-HA antibodies. Counterstaining was performed using Hoechst. Dashed circles indicate HA-JMJD1A-positive cells. Scale bar, 50 μm. (**C**) JMJD1A activity was determined by the *in vitro* demethylation assay in the presence or absence of DFO. Flag-Twin-Strep-tagged-WT-JMJD1A or Flag-Twin-Strep-tagged-H1122A-JMJD1A were affinity purified from 3T3-L1 preadipocytes overexpressing the respective proteins. Acquired purified WT-JMJD1A or H1122A-JMJD1A was reacted with the synthesized H3K9me2 peptide in a buffer (50 mM HEPES–KOH, pH 7.5, 1 mM α-KG, 2 mM ascorbic acid, and 70 μM ferrous ammonium sulfate) with or without 100 μM DFO (top). Flag-Twin-Strep-tagged-WT-JMJD1A WT or Flag-Twin-Strep-tagged-H1122A-JMJD1A was affinity purified from 3T3-L1 cells induced to differentiate with or without 100 μM DFO for 2 days. The resultant purified WT-JMJD1A or H1122A-JMJD1A was reacted with the synthesized H3K9me2 peptide in an iron-free buffer (50 mM HEPES–KOH, pH 7.5, 1 mM α-KG, and 2 mM ascorbic acid) (bottom). Protein levels were determined by performing immunoblot analysis using anti-H3K9me2 and anti-FLAG. Uncropped images are shown in Supplementary Figure S10. (**D**) Eight-week-old male mice were intraperitoneally injected with DFO (100 mg/kg BW/day) for 2 weeks under a high-fat, high-cholesterol diet, and H3K9me2 levels in epididymal WAT were determined by ChIP-qPCR (mean ± s.e.m. of three biological replicates). ChIP signals were presented as a percentage of input DNA. The two-tailed Student *t*-test was performed for statistical analysis. *P < 0.05, **P < 0.01

Next, the iron-dependent regulation of JMJD1A enzyme activity was analyzed. First, we performed *in vitro* H3K9 demethylation activity analysis using the HTRF system, and found that the activity of recombinant JMJD1A decreases as the concentration of iron added to the reaction is reduced, and furthermore, H3K9 demethylation activity is not observed in the absence of iron (Figure 5A). Thus, JMJD1A is dysfunctional under iron deficient conditions. In addition, the amount of unmethylated H3K9 (H3K9me0) produced by demethylation of H3K9me2 by JMJD1A is less than that produced from H3K9me1 in the low iron concentration range studied, when compared over the same reaction time (Figure 5A). These data indicate that the two steps of demethylation of H3K9me2 (i.e. H3K9me2 to H3K9me1, and H3K9me1 to H3K9me0) by JMJD1A (76) are both iron-dependent. Second, to confirm iron-dependent JMJD1A demethylation activity in 3T3-L1 cells, immunocytochemical analysis was performed under DFO(−) and DFO(+) conditions on Day 2. Immunocytochemistry of H3K9me2 showed that 3T3-L1 cells transiently transfected with HA-tagged JMJD1A showed demethylation of H3K9me2 under the DFO(−) condition, whereas such demethylation was not observed when treated with DFO [DFO(+) condition] (Figure 5B). These results suggest that JMJD1A demethylation of H3K9me2 in 3T3-L1 cells is iron-dependent. Finally, to more directly demonstrate that intracellular JMJD1A demethylates H3K9me2 in an iron-dependent manner, we investigated iron-dependent JMJD1A activity using an *in vitro* assay system in which purified JMJD1A from 3T3-L1 cells reacts with an artificially synthesized H3K9me2 peptide. Flag-Twin-Strep-tagged JMJD1A was affinity purified from 3T3-L1 preadipocytes using StrepTactin beads, and its demethylation activity against the H3K9me2 peptide was analyzed under an iron concentration of 70 μM. The results showed that purified JMJD1A demethylated the synthesized H3K9me2 peptide, but such demethylation was not observed when 100 μM DFO was added during the reaction (Figure 5C, top, lanes 2 and 3). In addition, purified Mut-JMJD1A (H1122A) did not demethylate the H3K9me2 peptide (Figure 5C, top, lanes 4 and 5). To further investigate the importance of the intracellular binding of iron to JMJD1A for its activity, we performed the *in vitro* H3K9me2 demethylation activity assay without adding any iron to the assay tube. As a result, WT-JMJD1A purified from 3T3-L1 cells induced to differentiate to Day 2 under the DFO(−) condition showed a reduced level of H3K9me2 compared with Mut-JMJD1A (H1122A) purified under the same condition (Figure 5C, bottom, lanes 2 and 4). However, no such demethylation was observed in WT-JMJD1A from 3T3-L1 cells induced to differentiate to Day 2 under the DFO(+) condition (Figure 5C, bottom, lanes 2 and 3). These results indicate that H3K9me2 demethylation occurred due to intracellular iron binding to JMJD1A. Thus, a certain amount of intracellular iron is essential for the demethylation of H3K9me2 by JMJD1A. Whereas we showed that iron is important for H3K9me2 demethylation at the *in vitro* and cellular levels, whether DFO administration also inhibits histone demethylation *in vivo* in mice remains unclear. Therefore, we performed intraperitoneal injections of DFO at 100 mg/kgBW/day for 2 weeks in mice fed a high-fat, high-sucrose diet, which showed higher H3K9me2 levels in the *Pparg* region in epididymal WAT compared with vehicle-treated control mice (Figure 5D). This is consistent with the finding that cultured 3T3-L1 cells showed higher H3K9me2 levels in the *Pparg* region upon DFO treatment, suggesting the presence of a similar iron-dependent epigenomic regulation mechanism in the WAT of living mice.

### TET2 regulates adipocyte differentiation in an iron-dependent manner

**Figure 6.**
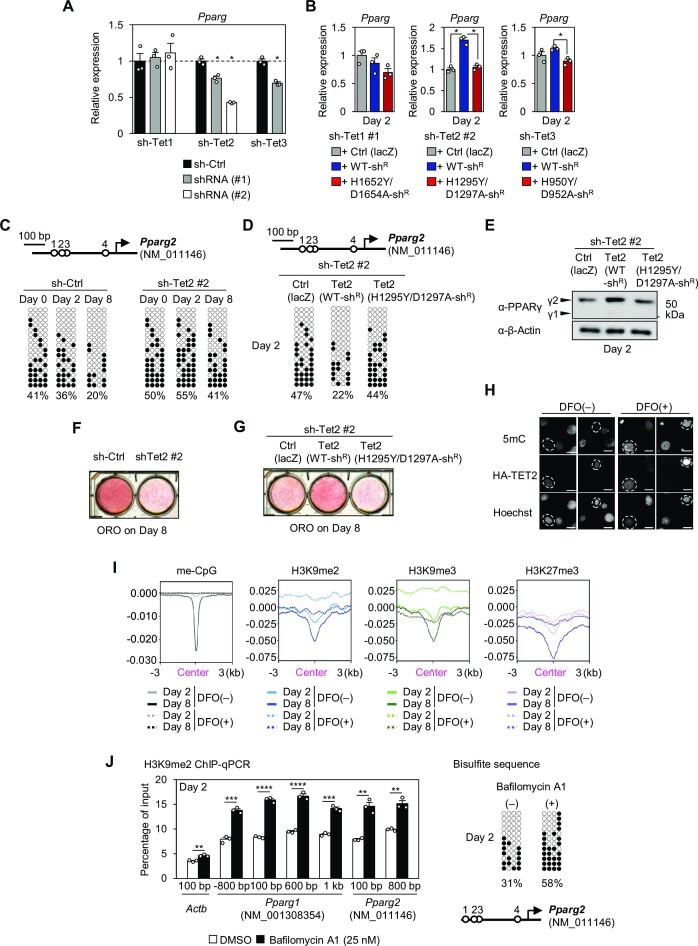
TET2 mediates iron-dependent DNA demethylation during adipocyte differentiation. (**A**) *Pparg* mRNA levels on Day 2 in a series of 3T3-L1 cell lines with knockdown of either TET1, TET2 or TET3 determined by qPCR (n = 3 biological replicates). The mRNA levels normalized to the *Cyclophilin B* mRNA level in the cell lines stably expressing shRNA for each target enzyme (shRNA #1 or shRNA #2) are shown as ratios to the level of the corresponding control cell line (sh-Ctrl). Data are shown as the mean ± s.e.m. The two-tailed Student's *t*-test was performed for the cell line stably expressing sh-Tet3. One-way ANOVA followed by the Dunnett test was performed for the cell lines stably expressing either sh-Tet1 or sh-Tet2. *P < 0.05. (**B**) The 3T3-L1 cell lines in which the expression of each demethylase was knocked down were retrovirally transduced with the corresponding enzyme with or without the indicated mutation in the iron-binding site. mRNA levels on Day 2 were measured by qPCR (*n* = 3 biological replicates). Data are shown as the mean ± s.e.m. One-way ANOVA followed by the Tukey-Kramer test was performed for statistical analysis. *P < 0.05. (**C**) Bisulfite sequence analysis of methylated CpGs in the promoter region of *Pparg2* (NM_011146) was performed using the TET2-KD cell line (sh-Tet2 #2) and its control cell line (sh-Ctrl) on Days 0, 2 and 8. The positions of the CpGs are shown at the top. Methylated and unmethylated CpGs are presented as closed and open circles, respectively (16 clones in each group). The levels of methylated CpGs under each condition are shown as percentages. (**D**) TET2-KD cell lines stably expressing either lacZ [Ctrl (lacZ)] or mouse TET2 harboring an shRNA-resistant mutation with (H1295Y/D1297A-sh^R^) or without (WT-sh^R^) additional mutations in the iron-binding site were differentiated into adipocytes. Bisulfite sequence analysis of methylated CpGs in the promoter region of *Pparg2* were performed as in (C). (**E**) TET2-KD cells (sh-Tet2 #2) overexpressing either lacZ [Ctrl (lacZ)], TET2 (WT-sh^R^), or TET2 (H1295Y/D1297A-sh^R^) were induced to differentiate, and protein levels of PPARγ and β-actin on Day 2 of differentiation were determined by immunoblot analysis using whole cell extracts. Uncropped images are shown in Supplementary Figure S10. (F, G) ORO staining of the cell lines presented in (C) and (D) was performed on Day 8 and presented in (**F**) and (**G**), respectively. (**H**) Cells transfected with pCAG-HA-Tet2 were cultured in the presence or absence of 100 μM DFO for 2 days, and immunostained with anti-5mC and anti-HA antibodies. Counterstaining was performed using Hoechst. Dashed circles indicate HA-TET2-positive cells. Scale bar, 20 μm. (**I**) Integrated analysis of WGBS data and ChIP-seq data of repressive histone marks (H3K9me2, H3K9me3 and H3K27me3) to determine the colocalization of histone demethylation and DNA demethylation. Aggregation plots showing changes in DNA and histone methylation levels around the DMRs that become less methylated during adipocyte differentiation. Methylation levels at the indicated time points compared with Day 0 are shown in the DMRs (±3 kb from center). (**J**) 3T3-L1 cells were induced to differentiate into adipocytes with 25 nM bafilomycin A1. ChIP-qPCR was performed using an anti-H3K9me2 antibody on Day 2 (mean ± s.e.m. of three biological replicates) (left). Bisulfite sequence analysis of methylated CpGs in *Pparg2* was performed on Day 0, 2 and 8 (right). The two-tailed Student's *t*-test was performed for statistical analysis. **P < 0.01, ***P < 0.001, ****P < 0.0001

Next, we investigated the iron-dependent effects of the Tet family of DNA demethylases. We found that knockdown of either TET2 or TET3 reduced *Pparg* expression (Figure 6A; Supplementary Figure S5A), and the latter was consistent with recent findings (77,78). Furthermore, in TET2-KD cells, the reduced *Pparg* expression was restored by the forced expression of TET2, which is the shRNA-resistant form of TET2 with a wild-type IBD (WT-TET2), but not by overexpression of the IBD mutant (Mut-TET2) containing both H1295Y and D1297A mutations, which prevent iron-dependent activity (Figure 6B; Supplementary Figure S5B). To analyze the iron-dependent DNA demethylation by TET2, DNA methylation levels in the *Pparg2* promoter region were determined (Figure 6C, D), as the *Pparg1* region is almost 100% methylated during the early stage of differentiation and hence cannot be increased any further (Supplementary Figure S5C). DNA methylation levels in the *Pparg2* region gradually decreased by half during adipocyte differentiation, from 41% to 20% (Figure 6C, left). However, TET2-KD cells showed 41% or higher levels of DNA methylation throughout the differentiation process (Figure 6C, right). The forced expression of WT-TET2 showed reduced DNA methylation levels on Day 2 in TET2-KD cells, but Mut-TET2 did not (Figure 6D). These changes in DNA methylation levels were inversely correlated with the levels of *Pparg* mRNA (Figure 6B) and PPARγ protein (Figure 6E) on Day 2 and lipid accumulation on Day 8 (Figure 6F, G). This restoration of DNA demethylation by WT-TET2 during the early stage of adipocyte differentiation may be due to modulation of its activity, as DFO treatment did not suppress *Tet2* expression during that period (Supplementary Figure S5D). Next, to confirm that iron-dependent TET2 demethylation occurs in 3T3-L1 cells, immunocytochemistry of 5mC was performed. Cells transiently transfected with HA-tagged TET2 showed lower 5mC levels under the DFO(−) condition, whereas such a change was not observed when cells were treated with DFO [DFO(+) condition] (Figure 6H). This data suggests that TET2 iron-dependently demethylates 5mC in 3T3-L1 cells.

### Cooperative regulation of adipogenesis by histone methylation and DNA methylation

In summary, both JMJD1A and TET2 were found to be involved in the regulation of *Pparg* expression. This suggests that these enzymes may cooperatively regulate adipocyte differentiation. In other words, histone methylation and DNA methylation are both altered in an iron-dependent manner, suggesting that they cooperatively regulate adipocyte differentiation. Therefore, we investigated the interrelationship between H3K9me2 and DNA methylation on a genome-wide basis by comparing WGBS with ChIP-seq data. Notably, we found that iron-dependent demethylation of H3K9me2 was often observed in DMRs (Figure 6I; Supplementary Figure S5E). Similar spatial interrelationships were observed between DMRs and iron-dependent demethylation of other repressive histone marks, namely, H3K9me3 and H3K27me3 (Figure 6I; Supplementary Figure S5E). To add further insight into the association between changes in DNA methylation and changes in histone modifications, data analysis of WGBS was performed by setting the putative enhancer regions based on previously reported H3K27ac data from Mikkelsen *et al.* (54) and DHS analysis data from Siersbæk *et al.* (55) (Supplementary Figure S6A). The results from both methods presented in Supplementary Figure S6A, B showed similar trends in the number of up/downregulated DMRs determined within the whole genome (Supplementary Figure S3H) and within regions 1 kb upstream of the TSS (Figure 3F). Namely, a significant number of DNA demethylated regions were observed only on Day 8 under the DFO(−) condition (Supplementary Figure S6A, B). Additionally, enrichment motif analysis of DNA demethylated enhancers showed high enrichment of CEBP, PPARE, RXR, and NF1 binding motifs (Supplementary Figure S6C), and this result was also similar to the results of analysis of the whole genome (Figure 3E). Iron-dependent DNA demethylation in enhancer regions during adipocyte differentiation was limited on Day 2 and apparent on Day 8, and was markedly suppressed by DFO treatment (Supplementary Figure S6D). This DNA demethylation change is even more pronounced when focusing only on enhancer regions that are activated during adipocyte differentiation (Supplementary Figure S6D). These data support the existence of a mechanistic link between histone methylation and DNA methylation during iron-dependent adipocyte differentiation. Consistently, the inhibition of iron supply by bafilomycin A1 treatment during the first two days of differentiation was found to increase both H3K9me2 and methylated DNA level in the *Pparg* genomic region on Day 2 (Figure 6J).

### Requirement of iron chaperon PCBP2 for epigenetic changes in adipocytes

**Figure 7.**
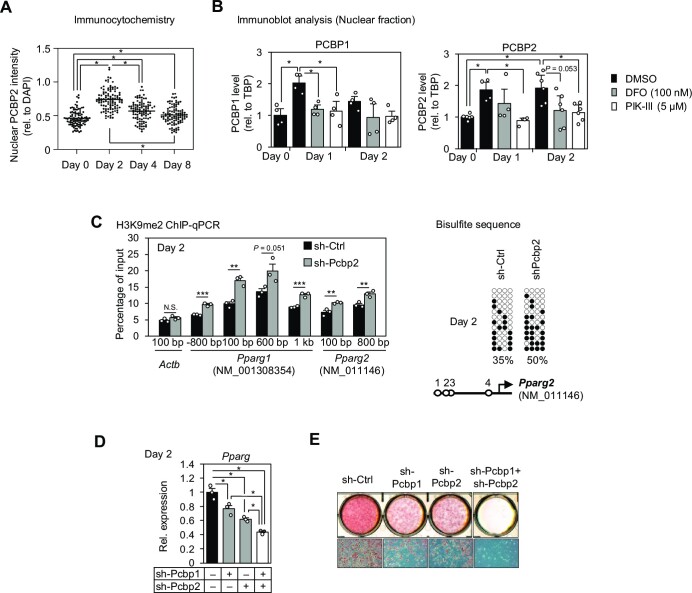
Iron chaperone PCBPs translocate to the nucleus and are crucial for adipocyte differentiation. (**A**) Nuclear PCBP2 levels in 3T3-L1 cells on Days 0, 2, 4 and 8 were quantified by immunostaining using an anti-PCBP2 antibody. The normalized PCBP2 fluorescence intensity to the DAPI signal is shown. The horizontal line indicates the median value of 100 nuclei. The Kruskal–Wallis test followed by the Steel–Dwass test was performed for statistical analysis. *P < 0.05. Microscopic images are shown in Supplementary Figure S7A. (**B**) The adipocyte differentiation of 3T3-L1 cells was induced together with the addition of either 100 μM DFO, 5 μM PIK-III, or DMSO. Nuclear fractions on the indicated day of differentiation were subjected to immunoblot analysis using anti-PCBP1, anti-PCBP2, and anti-TATA-binding protein (TBP) antibodies. Data are shown as fold change of the band intensities normalized to TBP (*n* = 4–6 biological replicates, as indicated in the dot plots). Data are shown as the mean ± s.e.m. One-way ANOVA followed by the Tukey-Kramer test was performed for statistical analysis. *P < 0.05, N.S., not significant. Uncropped immunoblot images are shown in Supplementary Figure S10. (**C**) ChIP-qPCR was performed using the anti-H3K9me2 antibody in PCBP2-KD cells (sh-Pcbp2) and control cells (sh-Ctrl) on Day 2 (mean ± s.e.m. of three biological replicates) (left). Bisulfite sequence analysis of methylated CpGs in the *Pparg2* promoter region were performed in PCBP2-KD cells and control cells on Day 2 (right). The two-tailed Student's *t*-test was performed for statistical analysis. **P < 0.01, ***P < 0.001. (**D**) *Pparg* mRNA levels on Day 2 in 3T3-L1 cell lines with single or double knockdown of PCBP1 and PCBP2, determined by qPCR (n = 3 biological replicates). *Pparg* mRNA levels normalized to *Cyclophilin B* level in the cell lines stably expressing shRNA (sh-Pcbp1, sh-Pcbp2, or both) are shown as ratios to the level of the corresponding control cell line (sh-Ctrl). Data are shown as the mean ± s.e.m. One-way ANOVA followed by the Tukey–Kramer test was performed for statistical analysis. *P < 0.05. (**E**) Differentiated 3T3-L1 cell lines with single or double knockdown of PCBP1 and PCBP2 were stained with ORO on Day 8. Scanned images of the cell culture plate (top) and bright-field microscopy images (bottom) are shown.

As both histones and DNA are localized in the nucleus, we speculated that intracellular iron transport to the nucleus is important for the demethylation of both histones and DNA during adipocyte differentiation. Therefore, we analyzed the translocation of the cytoplasmic iron chaperone, PCBPs, to the nucleus (Figure 7A, B). We first analyzed the nuclear levels of PCBP2, which binds a wider range of proteins than PCBP1 (9), and found that it is transiently increased on Day 2 (Figure 7A; Supplementary Figure S7A). This increase in nuclear PCBP2 level on Day 2 was suppressed by the administration of either DFO or PIK-III (Figure 7B, right). Similarly, nuclear PCBP1 levels also showed an increase, albeit more transient, during the first 2 days of differentiation, which was also suppressed by either DFO or PIK-III (Figure 7B, left). These data suggest that the nuclear translocation of PCPBs occurs in response to an increase in iron supply during the early stage of adipocyte differentiation. When PCBP2-KD cells were generated, both H3K9me2 and methylated DNA levels in the *Pparg* genomic region on Day 2 of these cells were higher than in control cells (Figure 7C). These findings collectively indicate that iron delivery to the nucleus by PCBP2 is required for the regulation of histone and DNA demethylation during the early stage of adipocyte differentiation. We further established and analyzed single and double knockdown cell lines of PCBP1 and PCBP2 (Supplementary Figure S7B), and found that *Pparg* expression was cooperatively suppressed in these cells (Figure 7D). In addition, double knockdown of both PCBP1 and PCPB2 markedly inhibited terminal adipocyte differentiation (Figure 7E). In summary, these results suggest that iron chaperone PCBPs are transported to the nucleus during the early stage of adipocyte differentiation, and regulate epigenomic changes during adipocyte differentiation.

## DISCUSSION

It has been reported that autophagy is essential for adipocyte differentiation, and is most active during the early stage (18–21). However, the importance of this time-specific induction of autophagy has rarely been recognized from the perspective of iron supply through the autophagic degradation of ferritin, namely, ferritinophagy. In the present study, we demonstrated that iron is essential for the early stage of adipocyte differentiation, and furthermore, that ferritinophagy is prominently activated during this period. These results suggest that iron demand is increased during early adipocyte differentiation, and that ferritin degradation is correspondingly accelerated to increase iron supply.

Sequential gene expression during adipocyte differentiation is regulated by both transcription factors and epigenetic mechanisms. Regarding the serial regulation of transcription factors, it is well known that C/EBPβ induces the expression of *Cebpa* and *Pparg*, which in turn activate each other's expression and regulate subsequent gene expression in a coordinated manner. Our results demonstrated that iron chelation by DFO suppresses the mRNA levels of *Cebpa* and *Pparg* but not *Cebpb*, indicating that iron is important for the expression of *Cebpa* and *Pparg*. It has been reported that autophagy promotes the transcription of *Cebpa* and *Pparg* by degrading Kruppel-like factor 2 (KLF2) and KLF3, which are negative regulators of adipocyte differentiation (18). In addition to this finding, we newly found that autophagy regulates *Cebpa* and *Pparg* transcription through iron-dependent epigenetic regulation. Regarding the epigenetic mechanisms of adipocyte differentiation, we noted that iron is essential for the activity of a series of epigenetic enzymes, including JmjC domain-containing histone demethylases and TET family DNA demethylases. As both histone demethylases and DNA demethylases affect gene transcription, we speculated that iron is involved in genome-wide transcriptional regulation. Therefore, we performed a genome-wide analysis of histone methylation and DNA methylation using next-generation sequencing, which demonstrated that iron-dependent histone demethylation and DNA demethylation occur during adipocyte differentiation. A comprehensive screen of epigenetic enzymes that regulate *Pparg* expression in an iron-dependent manner identified JMJD1A, JMJD2B, JHDM1D, PHF2 and PHF8. These are all H3K9 demethylases, and the enzyme that regulates H3K27 demethylation in an iron-dependent manner in the early stage of adipocyte differentiation was not identified in this study, and is hence the subject of future studies. For several histone demethylases, *Pparg* expression was reduced in their KD cells, but was not restored by forced expression of the corresponding gene. These results can be interpreted in light of previous reports that epigenetic enzymes regulate gene expression not only in an enzyme activity-dependent manner, but also in an enzyme activity-independent manner (25,79,80).

Additionally, TET2 and TET3 were identified as DNA demethylases that regulate *Pparg* expression in an iron-dependent manner, which is consistent with recent reports demonstrating that the latter is a regulator of adipocyte differentiation (77,78). These findings suggested that multiple enzymes cooperatively regulate transcription during adipocyte differentiation. Based on the integrated analysis of WGBS and ChIP-seq data, we found that the demethylation of repressive histone marks, including H3K9me2, H3K9me3, and H3K27me3, were frequently observed around the downregulated DMRs in the CpG regions, indicating a mechanistic link between histone methylation and DNA methylation. We also found that the overall trend of the demethylation of repressive histone marks was evident as early as Day 2, whereas genome-wide patterns of DNA demethylation were only very slightly observed on Day 2 and became apparent by Day 8 (Figure 6I, Supplementary Figure S5E). Because of the difference in the onset times of histone demethylation and DNA demethylation, we considered the possibility that histone demethylation precedes DNA demethylation. In the enhancer regions, a similar trend of iron-dependent DNA demethylation, which is very limited on Day 2 and becomes apparent on Day 8, was more pronounced when analyzed only in the regions that are activated during adipocyte differentiation (Supplementary Figure S6D). Considering that active enhancer marks and repressive histone marks are often mutually exclusive, these results are consistent with the hypothesis that the demethylation of repressive histone methylation modifications precedes the demethylation of DNA methylation modifications. It was previously reported that DNA methylation is a prerequisite for H3K9 methylation, when forming a bivalent chromatin domain consisting of H3K9me3 and H3K4me3 to maintain the preadipocyte state of 3T3-L1 cells (17). Together with this previous data, our present results suggest that the demethylation of methylated H3K9 precedes DNA demethylation during the resolution of the bivalent domain of H3K9me3 and H3K4me3 during adipocyte differentiation. However, more detailed studies are needed to reach this conclusion because of technical limitations due to the difference in sensitivity between ChIP-seq and WGBS, and because bisulfite sequencing cannot differentiate between 5mC and 5-hydroxymethylcytosine (5hmC).

The early stage of adipocyte differentiation is the time period when mitotic clonal expansion (MCE) of 3T3-L1 cells occurs. The number of cells counted during adipocyte differentiation was comparable between conditions with and without DFO addition on Day 1, but on Days 2 and 4, cell numbers decreased upon DFO treatment (Supplementary Figure S8A). Thus, DFO negatively affects MCE. In contrast, cell numbers of WT-JMJD1A-expressing JMJD1A-KD cells and Mut-JMJD1A-expressing JMJD1A-KD cells were comparable from Day 0 to Day 4 (Supplementary Figure S8B), and cell numbers of WT-TET2-expressing TET2-KD cells and Mut-TET2-expressing TET2-KD cells (Supplementary Figure S8C) showed the same trend. Although the detailed epigenomic mechanisms that regulate MCE in an iron-dependent manner are largely unknown, members of the KDM5 H3K4 demethylases (KDM5s) may play a role, because cell cycle-associated genes, such as *Cdc20*, *Plk1*, and *Ccna2*, that show reduced mRNA expression upon DFO treatment (Supplementary Figure S8D), are reported as direct targets of KDM5s (81). Based on the hypothesis that DFO suppresses the activity of KDM5s, which are demethylases of H3K4me3, it is assumed that H3K4me3 levels are maintained and target gene expression is increased; however, conversely, H3K4me3 levels in the genomic regions of cell cycle-associated genes and their mRNA expression are suppressed in cells treated with DFO [DFO(+)] compared with untreated cells [DFO(−)] (Supplementary Figure S8E). Considering the previous report that the KDM5s not only promotes but also represses target gene expression in an enzyme active site-dependent manner without necessarily mediating H3K4me3 (81), it is possible that other complex mechanisms besides the direct regulation of histone demethylation are involved in regulating cell cycle gene expression by DFO.

A possible mechanism for the upregulation of *Jmjd1a* and *Tet2* expression by DFO in the early stage of adipocyte differentiation shown in Figure 4H and Supplementary Figure S5D is the involvement of HIF-1α. This is because *Jmjd1a* expression has previously been reported to be induced by HIF-1α in several cell lines (e.g. endothelial and cancer cell lines) (82–84), and *Tet2* is also regulated by HIF-1α in HepG2 hepatocellular carcinoma cells (85). Additionally, as presented in Figure 2E, the HIF-1α signaling pathway is activated by DFO on Day 1. The fact that histone demethylation and DNA demethylation by JMJD1A and TET2, respectively, are suppressed by DFO treatment, whereas the expression of *Jmjd1a* and *Tet2* is increased by DFO treatment suggests that the activity of these enzymes is regulated by DFO.

Although the epigenomic mechanism by which specific iron-dependent enzymes regulate specific target genes is currently unknown, iron sensitivity may vary among enzymes or there may be context-dependent binding between enzymes and iron chaperones, such as PCBPs.

In addition to epigenetic enzymes, iron also binds in catalytic centers of other enzymes. Screening all such enzymes based on their effects on adipocyte differentiation is beyond the scope of this study. However, among these enzymes, mitochondrial aconitase, which requires iron-sulfur clusters (65), is an important iron-dependent enzyme in terms of its regulation of epigenetic enzyme activity. It is a constituent enzyme of the tricarboxylic acid (TCA) cycle and catalyzes the reversible isomerization of citrate to isocitrate. Because isocitrate is converted to α-KG in the TCA cycle, and α-KG is another cofactor essential for the activity of histone and DNA demethylases besides iron (16,86), the iron-dependent activation of mitochondrial aconitase may affect epigenetic demethylation. Indeed, intracellular and nuclear α-KG levels were increased during the early stage of adipocyte differentiation, but were suppressed by DFO treatment (24) (Supplementary Figure S9). This suggests that iron rewrites the epigenetic landscape during adipocyte differentiation by directly, and perhaps even indirectly, regulating the activity of epigenetic demethylases.

Taken altogether, our present study demonstrates that iron is essential during the early stage of adipocyte differentiation, and ferritinophagy is most active during this stage, to meet the increased iron demand. Iron is subcellularly transported by PCBPs to the nucleus during the early stage of adipocyte differentiation, and mediates adipocyte differentiation. An inadequate iron supply during the early stage of adipocyte differentiation disrupts the demethylation of repressive histone methylation marks and DNA methylation in the genomic regions of adipocyte differentiation-associated genes, resulting in suppression of the terminal differentiation of adipocytes. In particular, JMJD1A and TET2 are the major iron-dependent demethylases that regulate *Pparg* expression during the early stage of adipocyte differentiation. Thus, iron is indispensable for epigenetic rewriting during the adipocyte differentiation process. Whether physiological changes in iron levels epigenetically regulate gene expression in a dose-dependent manner during adipocyte differentiation *in vivo* remains unclear, and is a topic for future study.

## DATA AVAILABILITY

Next generation sequencing datasets reported in this study have been deposited to Gene Expression Omnibus with the accession numbers of GSE174136.

## Supplementary Material

gkad342_Supplemental_Files
