# Supplementary material for: Crucial role of iron in epigenetic rewriting during adipocyte differentiation mediated by JMJD1A and TET2 activity
Source: Nucleic Acids Res. 2023 May 9;51(12):6120–42. doi: 10.1093/nar/gkad342 (PMC10325906; doi:10.1093/nar/gkad342)
Supplement: gkad342_Supplemental_Files [file gkad342_supplemental_files.zip › Supplementary information (Suzuki et al) revision.pdf]

Crucial role of iron in epigenetic rewriting during adipocyte differentiation mediated by JMJD1A and TET2 activity

Tomohiro Suzuki, Tetsuro Komatsu, Hiroshi Shibata, Akiko Tanioka, Diana Vargas, Reika Kawabata-Iwakawa, Fumihito Miura, Shinnosuke Masuda, Mayuko Hayashi, Kyoko Tanimura-Inagaki, Sumiyo Morita, Junki Kohmaru, Koji Adachi, Masayuki Tobo, Hideru Obinata, Tasuku Hirayama, Hiroshi Kimura, Juro Sakai, Hideko Nagasawa, Hideyuki Itabashi, Izuho Hatada, Takashi Ito, and Takeshi Inagaki

Supplementary Information

Supplementary Tables S1-S5, S8, and S9
(Supplementary Tables S6 and S7 are in separate Excel-formatted files)
Supplementary Figures S1-S10

Supplementary Table S1. Oligonucleotides for construction of plasmids containing an shRNA cassette

shRNA	Primer	Sequence (palindromic sequences in capital letters)
shJhdm1d #1	Forward	5'-gatccGTGATCGAACTTCCAGATTTActcgagTAAATCTGGAAGTTCGATCACttttttg-3'
	Reverse	5'-aattcaaaaaaGTGATCGAACTTCCAGATTTActcgagTAAATCTGGAAGTTCGATCACg-3'
shJhdm1d #2	Forward	5'-gatccGTGGATTTGATGTCCCTATTActcgagTAATAGGGACATCAAATCCACttttttg-3'
	Reverse	5'-aattcaaaaaaGTGGATTTGATGTCCCTATTActcgagTAATAGGGACATCAAATCCACg-3'
shJmjd1a #1	Forward	5'-gatccGAAGTTCCTGAGCAAGTTATTctcgagAATAACTTGCTCAGGAACTTCttttttg-3'
	Reverse	5'-aattcaaaaaaGAAGTTCCTGAGCAAGTTATTctcgagAATAACTTGCTCAGGAACTTCg-3'
shJmjd1a #2	Forward	5'-gatccGTGCGAAGTTTCGTTGGATTTctcgagAAATCCAACGAAACTTCGCACttttttg-3
	Reverse	5'-aattcaaaaaaGTGCGAAGTTTCGTTGGATTTctcgagAAATCCAACGAAACTTCGCACg-3
shJmjd2a #1	Forward	5'-gatccGCGAACATCCTACGACGATATctcgagATATCGTCGTAGGATGTTCGCttttttg-3'
	Reverse	5'-aattcaaaaaaGCGAACATCCTACGACGATATctcgagATATCGTCGTAGGATGTTCGCg-3'
shJmjd2a #2	Forward	5'-gatccGAGCACGTTGATGAGTGGAATctcgagATTCCACTCATCAACGTGCTCttttttg-3'
	Reverse	5'-aattcaaaaaaGAGCACGTTGATGAGTGGAATctcgagATTCCACTCATCAACGTGCTCg-3'
shJmjd2b #1	Forward	5'-gatccGCAGCGATGGAAACTGAAATGctcgagCATTTCAGTTTCCATCGCTGCttttttg-3'
	Reverse	5'-aattcaaaaaaGCAGCGATGGAAACTGAAATGctcgagCATTTCAGTTTCCATCGCTGCg-3'
shJmjd2b #2	Forward	5'-gatccGCCCATGTGTACTTGGAAATTctcgagAATTTCCAAGTACACATGGGCttttttg-3'
	Reverse	5'-aattcaaaaaaGCCCATGTGTACTTGGAAATTctcgagAATTTCCAAGTACACATGGGCg-3'
shJmjd2c #1	Forward	5'-gatccGCAACCAAGAAGAGACATATTctcgagAATATGTCTCTTCTTGGTTGCttttttg-3'
	Reverse	5'-aattcaaaaaaGCAACCAAGAAGAGACATATTctcgagAATATGTCTCTTCTTGGTTGCg-3'
shJmjd2d #1	Forward	5'-gatccGCCACGGTAAGTAACGTTCCTctcgagAGGAACGTTACTTACCGTGGCttttttg-3'
	Reverse	5'-aattcaaaaaaGCCACGGTAAGTAACGTTCCTctcgagAGGAACGTTACTTACCGTGGCg-3'
shJmjd3 #1	Forward	5'-gatccGTTGAGCACAAACGGAACTATctcgagATAGTTCCGTTTGTGCTCAACttttttg-3'
	Reverse	5'-aattcaaaaaaGTTGAGCACAAACGGAACTATctcgagATAGTTCCGTTTGTGCTCAACg-3'
shJmjd3 #2	Forward	5'-gatccGCTGGATGAATCCATTCGGAActcgagTTCCGAATGGATTCATCCAGCttttttg-3'
	Reverse	5'-aattcaaaaaaGCTGGATGAATCCATTCGGAActcgagTTCCGAATGGATTCATCCAGCg-3'
shPhf2 #1	Forward	5'-gatccGAGCTGAAGATAGACGAGTTTctcgagAAACTCGTCTATCTTCAGCTCttttttg-3'
	Reverse	5'-aattcaaaaaaGAGCTGAAGATAGACGAGTTTctcgagAAACTCGTCTATCTTCAGCTCg-3'
shPhf8 #1	Forward	5'-gatccGCAAGATGAAACTCGGTGATTctcgagAATCACCGAGTTTCATCTTGCttttttg-3'
	Reverse	5'-aattcaaaaaaGCAAGATGAAACTCGGTGATTctcgagAATCACCGAGTTTCATCTTGCg-3'
shPhf8 #2	Forward	5'-gatccGCTGGCCAGTTGAGCTATAATctcgagATTATAGCTCAACTGGCCAGCttttttg-3'
	Reverse	5'-aattcaaaaaaGCTGGCCAGTTGAGCTATAATctcgagATTATAGCTCAACTGGCCAGCg-3'
shUtx #1	Forward	5’-gatccGCTACGAATCTCTAATCTTAActcgagTTAAGATTAGAGATTCGTAGCttttttg-3’
	Reverse	5’-aattcaaaaaaGCTACGAATCTCTAATCTTAActcgagTTAAGATTAGAGATTCGTAGCg-3’
shUtx #2	Forward	5’-gatccGCAGCACGAATTAAGTATTTActcgagTAAATACTTAATTCGTGCTGCttttttg-3’
	Reverse	5’-aattcaaaaaaGCAGCACGAATTAAGTATTTActcgagTAAATACTTAATTCGTGCTGCg-3’

Supplementary Table S1. Continued

shRNA	Primer	Sequence (palindromic sequence in capital letters)
shTet1 #1	Forward	5'-gatccGCAACTTGCATCCACGATTAActcgagTTAATCGTGGATGCAAGTTGCttttttg-3'
	Reverse	5'-aattcaaaaaaGCAACTTGCATCCACGATTAActcgagTTAATCGTGGATGCAAGTTGCg-3'
shTet1 #2	Forward	5'-gatccGTTTCAACTCCGACGTAAATActcgagTATTTACGTCGGAGTTGAAACttttttg-3'
	Reverse	5'-aattcaaaaaaGTTTCAACTCCGACGTAAATActcgagTATTTACGTCGGAGTTGAAACg-3'
shTet2 #1	Forward	5'-gatccGCTGGACATTTGTCTTGAAActcgagTTTCAAGACAAATGTCCAGCttttttg-3'
	Reverse	5'-aattcaaaaaaGCTGGACATTTGTCTTGAAActcgagTTTCAAGACAAATGTCCAGCg-3'
shTet2 #2	Forward	5'-gatccGAGCGTTCCTCAGTATCATTTctcgagAAATGATACTGAGGAACGCTCttttttg-3'
	Reverse	5'-aattcaaaaaaGAGCGTTCCTCAGTATCATTTctcgagAAATGATACTGAGGAACGCTCg-3'
shTet3 #1	Forward	5'-gatccGAACCTTCTCTTGCGCTATTTctcgagAAATAGCGCAAGAGAAGGTTCttttttg-3'
	Reverse	5'-aattcaaaaaaGAACCTTCTCTTGCGCTATTTctcgagAAATAGCGCAAGAGAAGGTTCg-3'
shFto #1	Forward	5'-gatccGACATCGAGACACCAGGATTActcgagTAATCCTGGTGTCTCGATGTCttttttg-3'
	Reverse	5'-aattcaaaaaaGACATCGAGACACCAGGATTActcgagTAATCCTGGTGTCTCGATGTCg-3'
shFto #2	Forward	5'-gatccGATGATGAAGTGGACCTTAAGctcgagCTTAAGGTCCACTTCATCATCttttttg-3'
	Reverse	5'-aattcaaaaaaGATGATGAAGTGGACCTTAAGctcgagCTTAAGGTCCACTTCATCATCg-3'
shPcbp1	Forward	5'-gatccGCATGATCCAACTGTGTAATTctcgagAATTACACAGTTGGATCATGCttttttg-3'
	Reverse	5'-aattcaaaaaaGCATGATCCAACTGTGTAATTctcgagAATTACACAGTTGGATCATGCg-3'
shPcbp2	Forward	5'-gatccGGCAATGCAACAGTCTCATTTctcgagAAATGAGACTGTTGCATTGCCttttttg-3'
	Reverse	5'-aattcaaaaaaGGCAATGCAACAGTCTCATTTctcgagAAATGAGACTGTTGCATTGCCg-3'
sh-Ncoa4	Forward	5'-gatccGATCTCATCTATCAGCTTAActcgagTTAAGCTGATAGATGAGATCttttttg-3'
	Reverse	5'-aattcaaaaaaGATCTCATCTATCAGCTTAActcgagTTAAGCTGATAGATGAGATCg-3'

Supplementary Table S2. Iron-binding site mutations in proteins used in this study

Genes	Source	Reference wild-type sequence	Iron-binding site mutations in translated proteins
Jhdm1d (encoding a.a. 71–940)	ES cells (B6 x 129 F1)	NM_001033430.4	H282A
Jmjd1a	ES cells (B6 x 129 F1)	NM_001038695.3	H1122A
Jmjd2b	ES cells (B6 x 129 F1)	NM_172132.3	H189A
Jmjd2c	ES cells (B6 x 129 F1)	NM_144787.2	H190A
Jmjd2d	ES cells (B6 x 129 F1)	NM_173433.2	H189A
Phf2	ES cells (B6 x 129 F1)	NM_011078.3	H249A
Phf8	ES cells (B6 x 129 F1)	NM_001113354.1	H247A
hUtx	Kazusa DNA Res. Inst.	KIBB3038	H1146A, E1148A
Tet1	ES cells (B6 x 129 F1)	NM_001253857.2	H1652Y, D1654A
Tet2	Kazusa DNA Res. Inst.	mKIAA7005	H1295Y, D1297A
Tet3	Kazusa DNA Res. Inst.	mKIAA7006	H950Y, D952A
Fto	3T3-L1 cells	NM_011936.2	H228A, D230A

Supplementary Table S3. Primers used for PCR-based-site-directed mutagenesis to confer shRNA-resistance

Gene	Primer sequence (shRNA resistant sequence in uppercase letter)	
Jhdm1d (encoding a.a. 71–940)	Forward primer	5'-AGCAGGTTCAATgctcaggacttaagtagaagccag-3'
	Reverse primer	5'-GGTCCTGTCGAActgtttattccaggaagaagtcatcaa-3'
Jmjd1a	Forward primer	5'-ACAGGTGATCcagtggcctgcaataatgtacaaatc-3'
	Reverse primer	5'-TCGGGCACCTCaggtgattttcgttctgccaaaac-3'
Jmjd2b	Forward primer	5'-AAGCTCAAGTGCatttactgccgcaagcgtatgaag-3'
	Reverse primer	5'-CCACCTCTGTTCagggatggcactgacgtc-3'
Jmjd2c	Forward primer	5'-TGGGCAGCAACgttgcctatatgtatcaggttgaatttgaag-3'
	Reverse primer	5'-GGTACTTGGCcccatagagtttaccatcaggccat-3'
Jmjd2d	Forward primer	5'-CAACGTGCCCtgcaacatgcaggtgtgcca-3'
	Reverse primer	5'-CTGACGGTGGCtataggcagaagagatcgtgagacc-3'
Phf2	Forward primer	5'-TCGACGAATTCcccatcaggaggaagaagagcg-3'
	Reverse primer	5'-TTTTCAGCTCcccatcatctgatacgtactcatactca-3'
Phf8	Forward primer	5'-GCTGGGCGACTTTgtcaaatactattacagtgggaagaggga-3'
	Reverse primer	5'-TTCATTTTGCAgtcagcctggcgggccaca-3'
hUtx	Forward primer	5'-CCTGATCCTCAAAgctgaaggaaaagtggagtctg-3'
	Reverse primer	5'-CTCTCATAGCAgcgaacagccttgccca-3'
Tet1	Forward primer	5'-GCACCATCAATcacatcaacgccgaggtca-3'
	Reverse primer	5'-TGGCCAGCTGggtcaattgtgctgcgacatct-3'
Tet2	Forward primer	5'-CAATACCACTTCcagcaaagagtaaatccctccagtg-3'
	Reverse primer	5'-GGGCACGCTGGGtgcttgcgggtgagcctc-3'
Tet3	Forward primer	5'-TGGCCCTCTTCgcacctagtccctccgggga-3'
	Reverse primer	5'-GGCTGGGCTCtggggttagaggggtggcac-3'
Fto	Forward primer	5'-GACTCCTGGCCTGacaatccctcttcaccagggag-3'
	Reverse primer	5'-TCGATGTCccaagagatcttaaaaccaac-3'

Supplementary Table S4. Primers for RT–qPCR

Gene	Forward sequence	Reverse sequence
Cyclophilin B (*Ppib*)	5'-GGAGATGGCACAGGAGGAA-3'	5'-GCCCGTAGTGCTTCAGCTT-3'
Pparg	5'-CACAATGCCATCAGGTTTGG-3'	5'-GCTGGTCGATATCACTGGAGATC-3'
Cebpa	5'-AGATGAGGGAGTCAGGCCGT-3'	5'-CGGAAAGTCTCTCGGTCTCAA-3'
Jmjd1a	5'-CATGATGCCTTCCAGGTTTG-3'	5'-GCCATCTCGCCTGGTGTACT-3'
Jhdm1d	5'-AGCTCTACACGGCTCTTCCTTAAT-3'	5'-AGCTCTACACGGCTCTTCCTTAAT-3'
Phf2	5'-CCTATTGGAAGCGTTCAAAGG-3'	5'-TAAAAGCACCATTGAGAATTTTAGCT-3'
Phf8	5'-TCTCCGATACCAGGCTTTCAA-3'	5'-CCATGACAGCTTGCGAACAA-3'
Jmjd3	5'-AAAGACGAGCCTGCCTACTACTG-3'	5'-TGCCATTCTCACTTGTAACGAA-3'
Jmjd2a	5'-GCATGCTAGTGTCCTGCAAGA-3'	5'-GGAGGGACGCCATAGCAA-3'
Jmjd2b	5'-GCCACACAGTGTACATGTCGAA-3'	5'-GCCTTGCTTCCATTGCTCAT-3'
Jmjd2c	5'-GGAGCCAAACTGTGCCATCT-3'	5'-TTCTTTGCTGCTATCAGGCTTGT-3'
Jmjd2d	5'-GGTCACCTGGGAACAATTCAA-3'	5'-TGACGCCCTCAATCACTATGC-3'
Tet1	5'-TTGAGGTCCTACAGCGGACAT-3'	5'-TGCAGGTACGCTTTTTGTTGA-3'
Tet2	5'-GCTCTCAGGGATGTCCTATTGC-3'	5'-TGATTAGGTCGCACTCGTACCA-3'
Tet3	5'-GATCTGGCCCCACAGTAGCTT-3'	5'-CCCTTTTCTCCATACCGATCCT-3'
hUtx	5'-GGCATGGGCGGACAA-3'	5'-AGATCAAAAACCTCCACTTCACA-3'
Fto	5'-CTGTGGAAGAAGATGGAGAGCAT-3'	5'-CGGGAGCCCCTCTCTTTTAA-3'
Pcbp1	5'-CAGATCTGCCTGGTCATGCT-3'	5'-GCTGGTACGGGATGGTCAT-3'
Pcbp2	5'-CACTTTGGCTGGACCGACTA-3'	5'-TGCTTATGTCCTCTTCCAGTTTG-3'
Ncoa4	5'-GGTTGATAAAGAAAAATGGCTGTACA-3'	5'-GCTGCTCGATTCTGGTAGTCTGT-3'

Supplementary Table S5. Antibodies used in this study

Antibody	Monoclonal/ polyclonal	Source	Concentration/ amount	Catalog number/ clone number
anti-Ferritin	Monoclonal	Abcam	1:2,000 (34 ng/mL) for IB<br>1:2,00 (340 ng/mL) for IS	ab75973/EPR3004Y
anti-PPARγ	Monoclonal	Cell signaling Technology	1:2,000 (149 ng/mL) for IB	#2435/C26H12
anti-C/EBPα	Monoclonal	Cell signaling Technology	1:2,000 (10.4 ng/mL) for IB	#8178/D56F10
anti-NCOA4	Polyclonal	Bethyl Laboratories	1:2,000 (500 ng/mL) for IB<br>1:200 (5 μg/mL) for IS	A302-272A
anti-LC3	Polyclonal	MBL Life Science	1:4,000 (1.25 μg/mL) for IB	PM036
	Monoclonal	MBL Life Science	1:200 (10 μg/mL) for IS	M152-3/4E12
anti-PCBP1	Polyclonal	Cell signaling Technology	1:2,000 (71.5 ng/mL) for IB<br>1:1,000 (143 ng/mL) for IS	#8534
anti-PCBP2	Monoclonal	Cell signaling Technology	1:2,000 (55.5 ng/mL) for IB<br>1:1,000 (111 ng/mL) for IS	#83017/D1S5E
anti-TfR	Monoclonal	Invitrogen	1:2,000 (250 ng/mL) for IB	#13-6800/H68.4
anti-β-Actin	Monoclonal	Cell signaling Technology	1:2,000 (30.5 ng/mL) for IB	#4970/13E5
	Monoclonal	Cell signaling Technology	1:2,000 (420 ng/mL) for IB	#3700/8H10D10
anti-GAPDH	Monoclonal	Cell signaling Technology	1:5,000 (12.4 ng/mL) for IB	#5174/D16H11
anti-FLAG	Monoclonal	Sigma	1:1000 (4.3 μg/mL) for IB	F3165/M2
anti-H3K4me3	Polyclonal	Merck	1 μg per IP for ChIP	#07-473
anti-H3K9me2	Monoclonal	Dr. Kimura	1:200 (10 μg/mL) for IS<br>2 μg per IP for ChIP	CMA317/6D11
anti-H3K9me2-HRP	Monoclonal	HRP was conjugated to anti-H3K9me2 from Dr. Kimura	400 ng/mL for IB	CMA317/6D11
anti-H3K9me3	Monoclonal	DOJINDO	1:20 (Concentration info. unavailable) for IS	H419
	Monoclonal	Dr. Kimura	5 μg per IP	CMA318/2F3
anti-H3K27me3	Monoclonal	DOJINDO	1:50 (Concentration info. unavailable) for IS	H421
	Monoclonal	Dr. Kimura	2.5 μg per IP	CMA323/1E7
anti-H3K27ac	Monoclonal	DOJINDO	1:50 (Concentration info. unavailable) for IS	H422
anti-HA	Polyclonal	SantaCruz	1:20 (5 μg/mL) for IS	Y-11
anti-5mC	Monoclonal	Abcam	1:500 (2 μg/mL) for IS	ab10805/33D3

IB: immunoblotting, IS: immuno-staining, ChIP: chromatin immunoprecipitation

Supplementary Table S8. Primers for ChIP–qPCR

Gene	Reference transcript	Position from TSS	Primers	
Actb	NM_007393.5	+100 bp	Forward	5'-TGAGGTACTAGCCACGAGAGAG-3'
			Reverse	5'-ACACCCGCCACCAGGTAAGCA-3'
Pparg1	NM_001308354	–800 bp	Forward	5'-CTGCGTAACTGACAGCCTAAC-3'
			Reverse	5'-ACTTGGTCACTCTCCGTCCT-3'
		+100 bp	Forward	5’-TGTGTGTAGTGAAGTGGCCAAAC-3’
			Reverse	5’-ACACAGCCAGACCATCCTGAT-3’
		+600 bp	Forward	5’-TCCTGTCGTCCATAGCTAAGTGATC-3’
			Reverse	5’-CCCTAGCCAGGTTTTAAATCACA-3’
		+1 kbp	Forward	5’-CTTCAGTGAGAAGATCTAAGGAGCAA-3’
			Reverse	5’-TCCCTTCTAGTGAGCTGCAAAAG-3’
Pparg2	NM_011146	+100 bp	Forward	5'-CTGGGAGATTCTCCTGTTGACC-3'
			Reverse	5'-CCTTGCAGCAACATCAGGAA-3'
		+800 bp	Forward	5’-CAGATTTGCTTCACGGGAACA-3’
			Reverse	5’-TGCAACATTTACAGTGGACAAGAA-3’

Supplementary Table S9. Primers for DNA methylation assay and reference genome sequences before bisulfite conversion

Target	Sequence name	Forward sequence †	Reverse sequence †
Pparg1	Bisulfite primers	5'-GGGTTTATTAGGTGTGAGGAAAG-3'	5'-CTTCAAATCCCCAACAAAAATC-3'
	Reference genome sequence	5'-GGGCTCACCAGGTGTGAGGAAAG-3'<br>(coding sequence)	5'-CTTCAGATCCCCAGCAGAGGTC-3'<br>(complementary sequence)
Pparg2	Bisulfite primers	5'-TGGTAATTTTAATTATTGTATAGTTTA-3'	5'-ATTCACACTAATATTTTATCTATATCTTAC-3'
	Reference genome sequence	5'-TGGTAATTCCAACTACTGTACAGTTCA-3'<br>(coding sequence)	5'-ATTCACACTGGTGTTTTGTCTATGTCTTGC-3'<br>(complementary sequence)
M13	Insert PCR primers	5'-GTAAAACGACGGCCAG-3'	5'-CAGGAAACAGCTATGAC-3'

† Underlined nucleotides converted from the reference genome sequences for bisulfite primers

Supplementary Figure S1

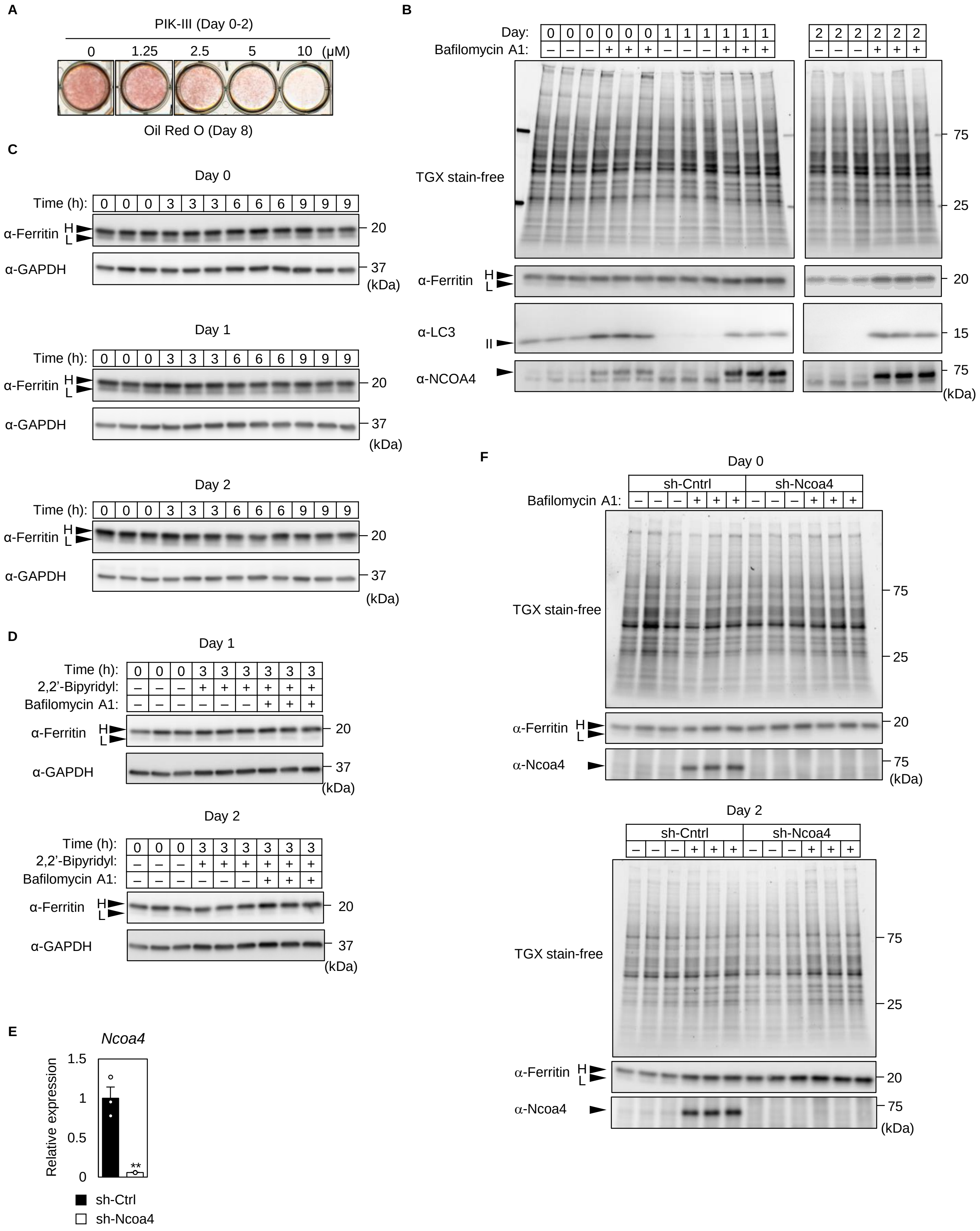

Supplementary Figure S1. Analysis of ferritinophagy during adipocyte differentiation. (**A**) 3T3-L1 cells were differentiated with PIK-III at the indicated concentrations for the first 2 days, and stained with ORO on Day 8. (**B**) The lysosomal fluxes of ferritin, LC3-II, and NCOA4 were measured by immunoblot analysis. 3T3-L1 cells were treated with 100 nM bafilomycin A1 or DMSO for 24 hours before preparing whole cell lysates, and then whole cell lysates were prepared on Days 0, 1, and 2 of differentiation. Samples were subjected to immunoblot analysis using anti-ferritin, anti-LC3, and anti-NCOA4 antibodies. The total protein levels were determined by TGX stain-free gel as a control. (**C**) 3T3-L1 cells on Days 0, 1, and 2 of differentiation were treated with 200 μM 2,2-bipyridyl for the indicated time periods (0, 3, 6, and 9 hours), and then whole cell lysates were prepared at each time point. Samples were subjected to immunoblot analysis using anti-ferritin and anti-GAPDH antibodies. (**D**) 3T3-L1 cells on Days 1 and 2 of differentiation were treated with 200 μM 2,2-bipyridyl in the presence of 100 nM bafilomycin A1 or DMSO for 3 hours, and then whole cell lysates were prepared for immunoblot analysis using anti-ferritin and anti-GAPDH antibodies. H and L (**B**–**D**), and I and II (**B**) with arrow heads indicate positions of heavy and light chains of Ferritin, and those of LC3-I and LC3-II, respectively. (**E**) Knockdown efficiency of Ncoa4 knockdown cells (sh-Ncoa4) is shown as a ratio to the level of the corresponding control cell line (sh-Ctrl) (n = 3). Data are shown as the mean ± s.e.m. The two-tailed Student‘s *t*-test was performed for statistical analysis. ***P* < 0.01. (**F**) The lysosomal fluxes of ferritin and NCOA4 in Ncoa4-KD cells were measured by immunoblot analysis. The cells were treated with 100 nM bafilomycin A1 or DMSO for 24 hours before preparing whole cell lysates, and then whole cell lysates were prepared on Days 0 and 2 of differentiation. Immunoblot analysis was performed using anti-ferritin and anti-NCOA4 antibodies. The total protein levels were determined by TGX stain-free gel as a control. Uncropped images of (B–D, F) are shown in Supplementary Figure S10.

Supplementary Figure S2

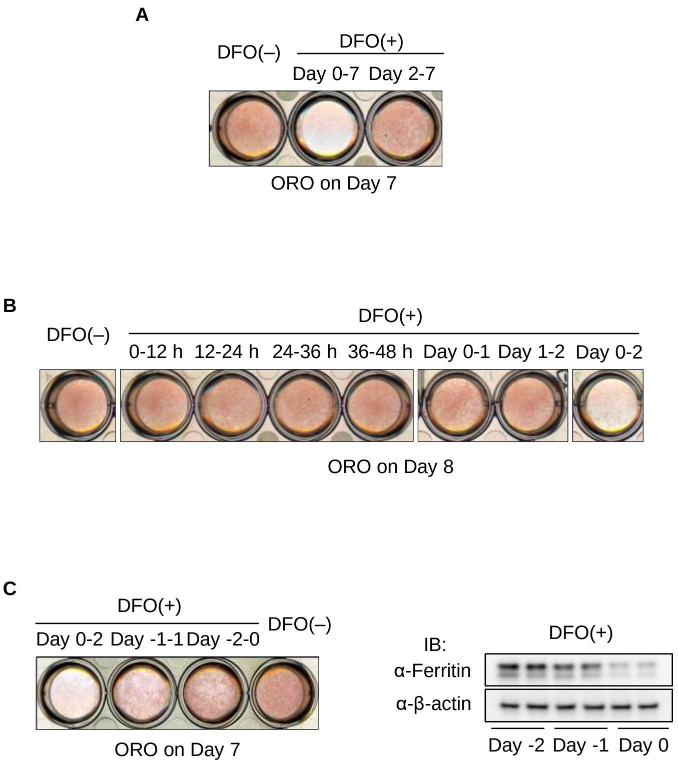

Supplementary Figure S2. The effect of iron deprivation at different periods during adipocyte differentiation. (**A**–**C**) 3T3-L1 cells were treated with vehicle (–) or DFO (100 μM) for the indicated periods during adipocyte differentiation. Cells were stained with ORO on Day 7 (A and C, left) or Day 8 (B) of differentiation. (C, right) The whole cell lysate was prepared on Day -2, Day -1, and Day 0 of differentiation, and immunoblot analysis was performed using anti-ferritin and anti-β-actin antibodies. Uncropped images of (C) are shown in Supplementary Figure S10.

Supplementary Figure S3

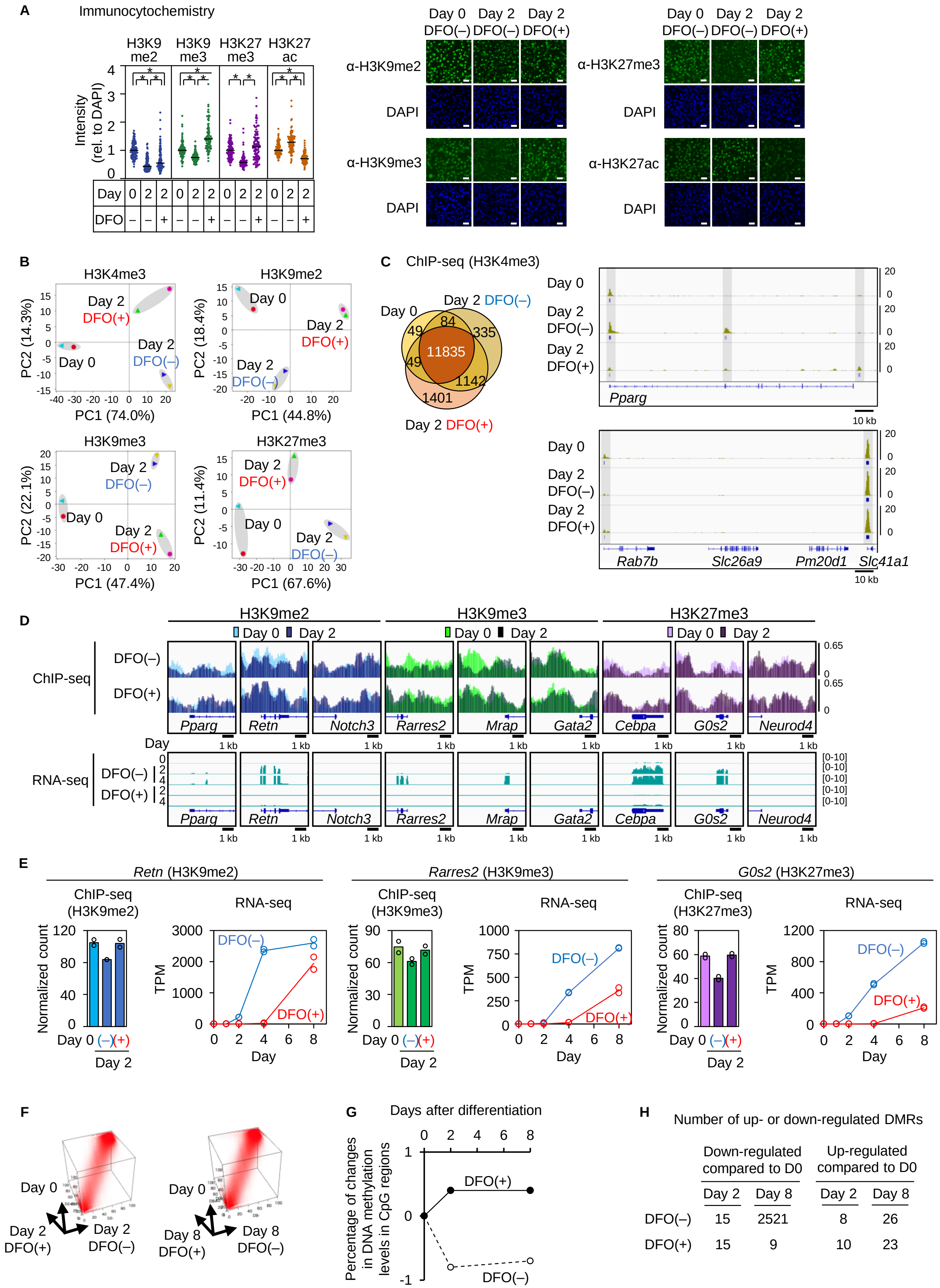

Supplementary Figure S3. Next generation sequencing analysis of methylated histone modifications, DNA methylation, and m^{6}A methylation during adipocyte differentiation. 3T3-L1 cells were differentiated into adipocytes with or without DFO (100 μM) for the first 2 days and the following analyses were performed. (**A**) Immunocytochemistry of histone modifications using anti-H3K9me2, anti-H3K9me3, anti-H3K27me3, and anti-H3K27ac antibodies and DAPI. The fluorescence levels of histone modifications were normalized to the DAPI levels. The median of the normalized fluorescence intensity of one hundred nuclei in each group is shown as a horizontal line. The Kruskal–Wallis test followed by the Steel–Dwass test was performed for statistical analysis. **P* < 0.05. Scale bar, 40 μm. (**B**) Principal component analysis (PCA) on ChIP-seq datasets for H3K4me3, H3K9me2, H3K9me3, and H3K27me3. Each replicate is presented as a colored symbol. Two replicates belonging to the same group is highlighted with a gray background. (**C**) Analysis of H3K4me3 ChIP-seq data. The Venn diagram depicting the number of H3K4me3 peaks for each condition determined by peak calling (left) and representative tracks of H3K4me3 peaks detected in two genomic loci (right). H3K4me3 signals are shown in dark yellow, together with peak positions indicated below by dark blue bars (right). (**D**) Representative tracks of ChIP-seq for the indicated histone marks and RNA-seq at the loci of genes in cluster 6 (*Pparg*, *Retn*, *Rarres2*, *Mrap*, *Cebpa*, and *G0s2*) and of genes with low expression (*Notch3*, *Gata2*, and *Neurod4*). Superimposed images of ChIP-seq signals before (Day 0; shown in light colors) and after (Day 2; shown in dark colors) induction of differentiation with or without DFO, to compare changes (top) and RNA-seq data (bottom) on the indicated day of differentiation with or without DFO treatment. (**E**) The normalized CPM of H3K9me2, H3K9me3, and H3K27me3 ChIP-seq signals in the TSS ± 5 kb of representative Cluster 6 genes in Fig. 3A (left). Transcriptional changes (TPM) of the genes determined by RNA-seq are shown (right). The circles represent two individual replicates for each treatment. (**F**–**H**) Analysis of WGBS data. Three-dimensional scatter plots showing methylation levels (%) at all CpG sites in the genome, compared before and after differentiation induction with or without DFO on Day 2 (F, left) or Day 8 (F, right). Changes in genome-wide CpG methylation levels (%) from Day 0 (G). The number of DMRs that significantly increased or decreased on Day 2 or 8 of differentiation, detected using the metilene software (H).

Supplementary Figure S4

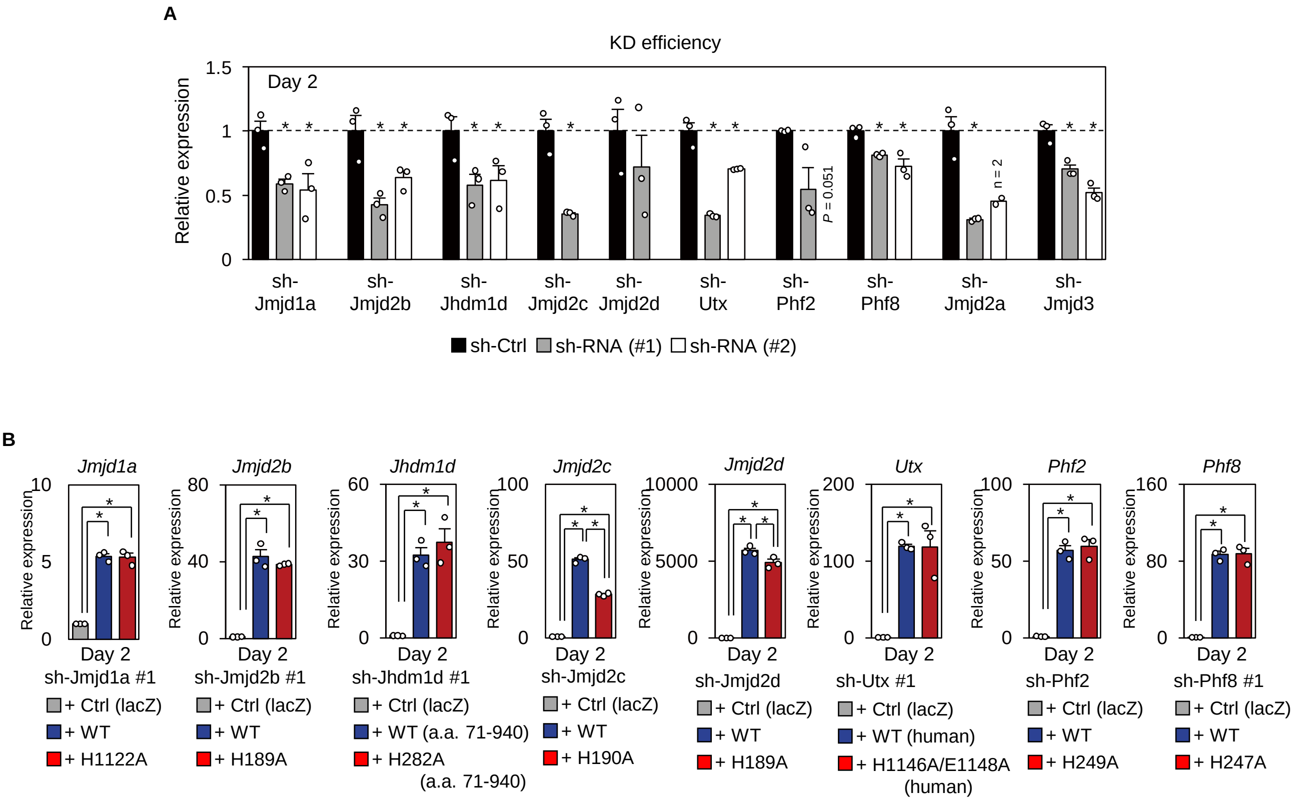

Supplementary Figure S4. Validation of knockdown and overexpression levels of target histone demethylases. (**A**) Knockdown efficiency of cell lines established by the stable expression of shRNA for each target enzyme (shRNA (#1) or shRNA (#2)) is shown as a ratio to the level of the corresponding control cell line (sh-Ctrl) (n = 3, unless otherwise specified). Data are shown as the mean ± s.e.m. The two-tailed Student's *t*-test was performed for the cell lines expression either sh-Jmjd2, sh-Jmjd2d, or sh-Phf2. One-way ANOVA followed by the Dunnett test was performed for the other cell lines. *$P < 0.05$. (**B**) The 3T3-L1 cell lines, in which the expression of each demethylase was knocked down, were retrovirally transduced the corresponding enzyme with or without the indicated mutation in the iron-binding sites. The mRNA levels of demethylase genes on Day 2 of differentiation were measured by qPCR (n = 3). The full-length mouse sequence was used for overexpression, except that the partial sequence encoding amino acid (a.a.) 71-940 was used for Jhdm1d and the human version was used for Utx. Data are shown as the mean ± s.e.m. One-way ANOVA followed by the Tukey-Kramer test was performed for statistical analysis. *$P < 0.05$.

Supplementary Figure S5

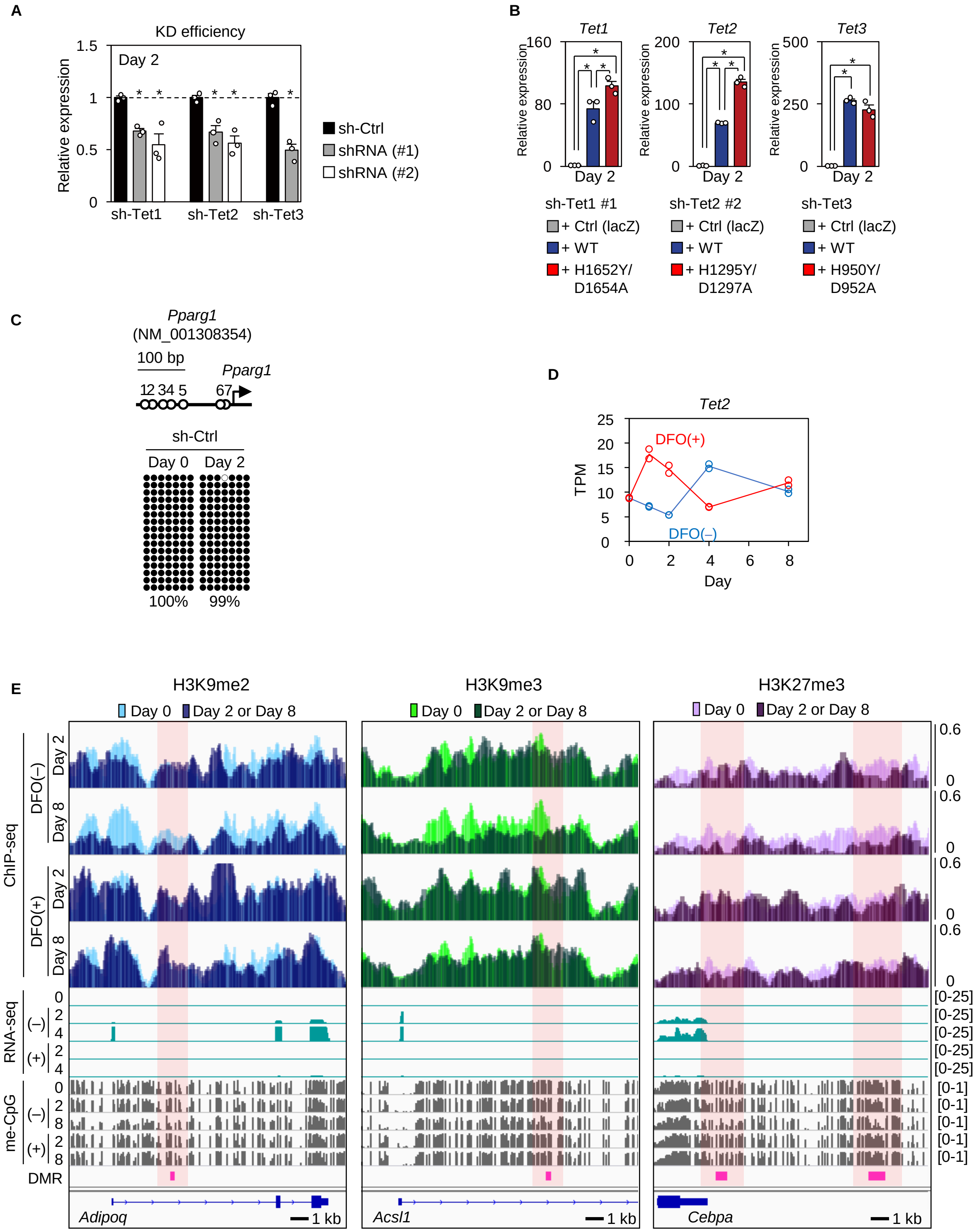

Supplementary Figure S5. The expression levels of DNA and RNA demethylases in their KD cell lines and the analysis of interrelationship between methylated histones and methylated DNA. (**A**) Knockdown efficiency of cell lines established by the stable expression of shRNA for each TET enzyme (shRNA (#1) or shRNA (#2)) is shown as a ratio to the level of the corresponding control cell line (sh-Ctrl) (n = 3). Data are shown as the mean ± s.e.m. The two-tailed Student's *t*-test was performed for the cell line expressing sh-Tet3. One-way ANOVA followed by the Dunnett test was performed for the cell lines expressing either sh-Tet1 or sh-Tet2. *$P < 0.05$. (**B**) The 3T3-L1 cell lines, in which the expression of each TET enzyme was knocked down, were retrovirally transduced the corresponding enzyme with or without the indicated mutation in the iron-binding sites. The mRNA levels of demethylase genes on Day 2 of differentiation were measured by qPCR (n = 3). Data are shown as the mean ± s.e.m. One-way ANOVA followed by the Tukey-Kramer test was performed. *$P < 0.05$. (**C**) Bisulfite sequence analysis of methylated CpGs in the regions of *Pparg1* was performed on Days 0 and 2 in control cells (sh-Ctrl). (**D**) Transcriptional changes (TPM) of *Tet2* determined by RNA-seq. The circles represent two individual replicates for each treatment. (**E**) Representative tracks of the methylated CpGs (me-CpG) determined by WGBS (gray bars, methylation levels shown in percent) and the ChIP-seq data for the indicated histone marks (H3K9me2, H3K9me3 or H3K27me3) at *Adipoq*, *Acsl1*, and *Cebpa* loci. Images of ChIP-seq signals before (Day 0; shown in light colors) and after (Day 8; shown in dark colors) induction of differentiation with or without DFO are overlayed in order to compare the changes. DMR positions are shown in dark pink bars, and the light pink area shows the regions of interest around the DMRs.

Supplementary Figure S6

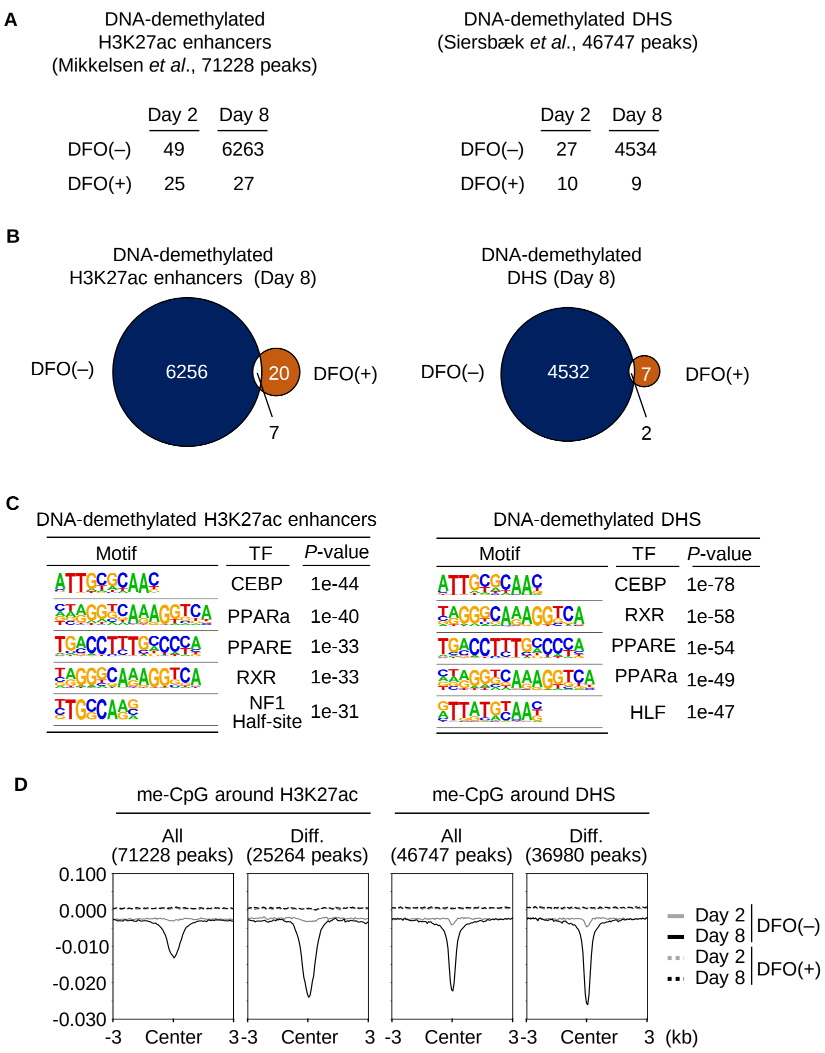

Supplementary Figure S6. WGBS data analysis within enhancer regions

Analysis of WGBS was performed by setting the putative enhancer regions based on previously reported H3K27ac ChIP-seq peaks (Mikkelsen *et al.* 2010 Cell) or DNase I hypersensitive sites (DHSs, Siersbæk *et al.* 2011 EMBO J.). (**A**) The analysis of DNA-demethylated enhancers ($\pm$ 0.5 kb from peak center). CpGs that differ in methylation level by more than 50% on Day 8 [DFO(–) or DFO(+)] compared to Day 0 were detected, and the number of enhancers with three or more such CpGs is shown. (**B**) The number of DNA-demethylated enhancers on Day 8 under DFO(–) or DFO(+) conditions is depicted in the Venn diagrams. (**C**) Motif analysis was conducted with DNA-demethylated H3K27ac enhancers and DHS on Day 8 under DFO(–) condition; all peaks of H3K27ac and DHS were used as background regions, respectively. (**D**) Changes in DNA methylation levels relative to Day 0 were analyzed using all enhancers (All) or enhancers that are activated only after the induction of differentiation (Diff.) are displayed as aggregation plots. The regions within 3 kb up- and down-stream of each peak center of putative enhancers are shown.

Supplementary Figure S7

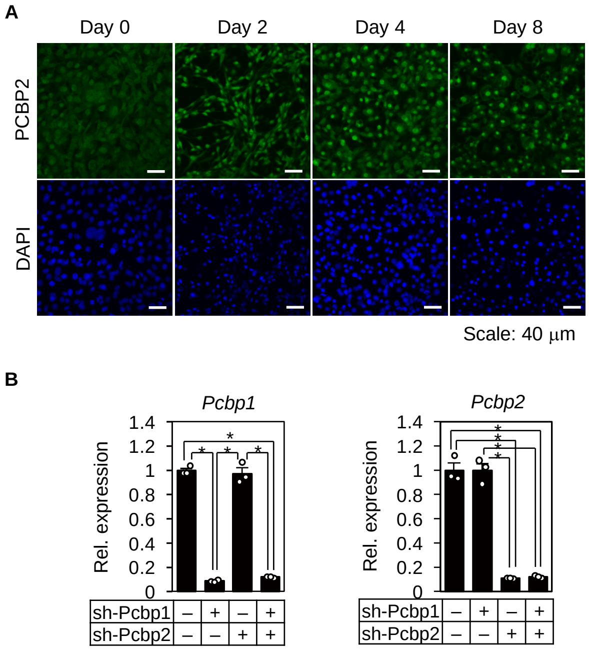

Supplementary Figure S7. Nuclear levels of PCBP2 during adipocyte differentiation. (**A**) Immunocytochemistry using anti-PCBP2 antibody and DAPI staining in 3T3-L1 cells on Days 0, 2, 4, and 8 of differentiation. (**B**) Knockdown efficiency of cell lines established by the stable expression of shRNA for Pcbp1 and/or Pcbp2 is shown as a ratio to the level of the corresponding control cell line (sh-Ctrl) (n = 3). Data are shown as the mean ± s.e.m. One-way ANOVA followed by the Tukey-Kramer test was performed for statistical analysis.

Supplementary Figure S8

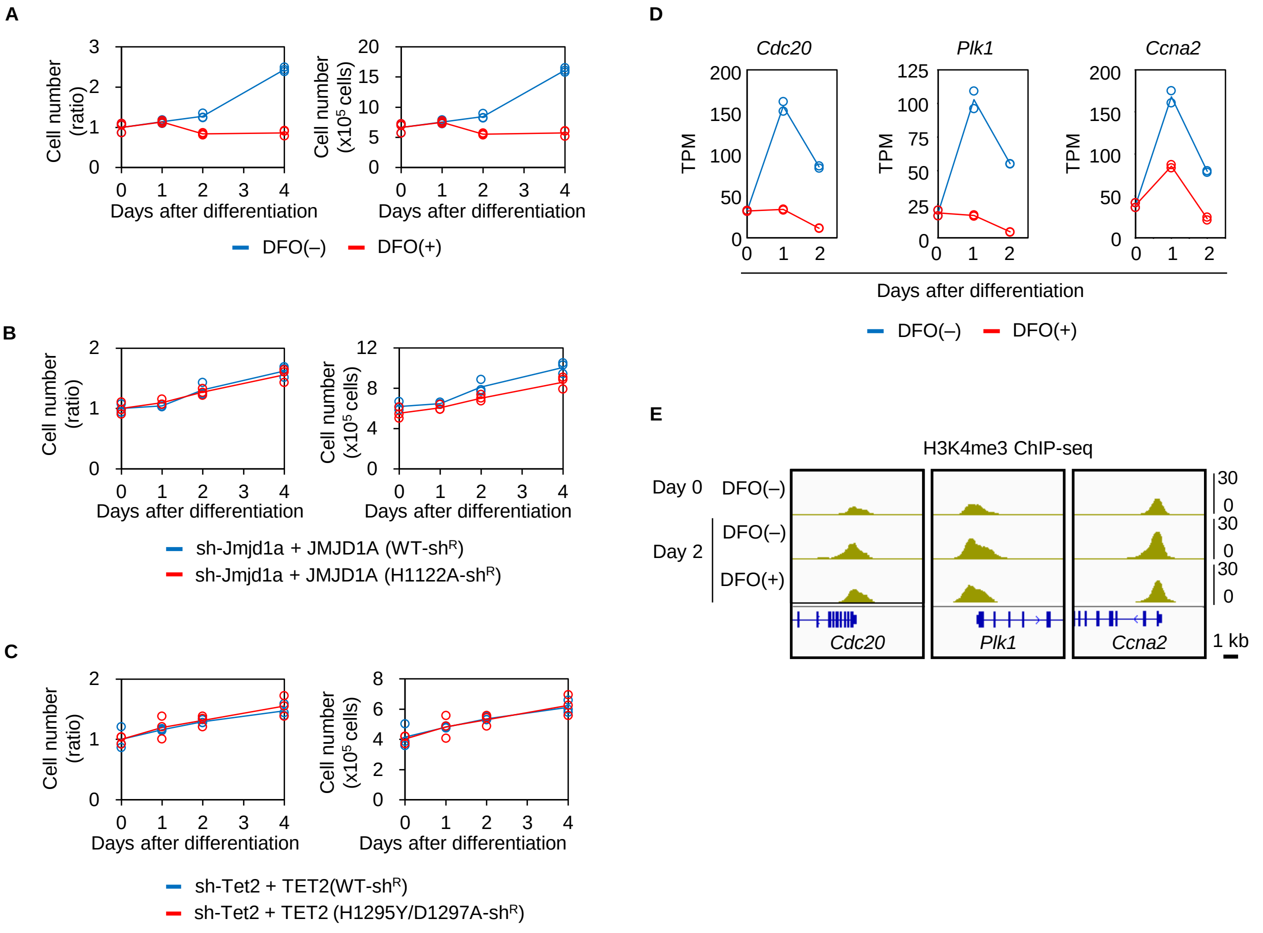

Supplementary Figure S8. MCE analysis and H3K4me3 levels in cell cycle-related genes
(**A**–**C**) Cell numbers after the induction of adipocyte differentiation was compared between 3T3-L1 cells differentiated with and without DFO (100 μM) from Day 0 to Day 2 (A), WT-JMJD1A-expressing Jmjd1a-KD cells and Mut-JMJD1A-expressing Jmjd1a-KD cells (B), and WT-TET2-expressing Tet2-KD cells and Mut-TET2-expressing Tet2-KD cells (C). The vertical axes show the ratio of the number of cells to that of Day 0 (left) and the actual number (right). The circles represent three biological replicates. (**D**) Transcriptional changes of cell cycle-related genes (*Cdc20*, *Plk1*, and *Ccna2*) by RNA-seq were shown in TPM. The circles represent two individual replicates for each treatment. (**E**) ChIP-seq profiles for H3K4me3 at the genomic loci of cell cycle-related genes (*Cdc20*, *Plk1*, and *Ccna2*). H3K4me3 signals are shown in dark yellow.

Supplementary Figure S9

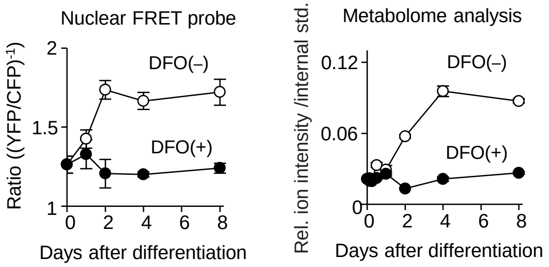

Supplementary Figure S9. Nuclear levels of α-KG during adipocyte differentiation. 3T3-L1 cells were treated with or without 100 μM DFO for the first 2 days during differentiation. Nuclear α-KG concentrations were determined in 3T3-L1 cells expressing the nuclear FRET-based α-KG probe and shown as the reciprocal of the FRET ratios (CFP/YFP) (left). Cellular α-KG concentrations in 3T3-L1 cells measured by LC-MS/MS are presented as relative ion intensities normalized to the internal standard (right).

Supplementary Figure S10

Figure 1B, 1D, and 1E, and Supplementary Figure 1B

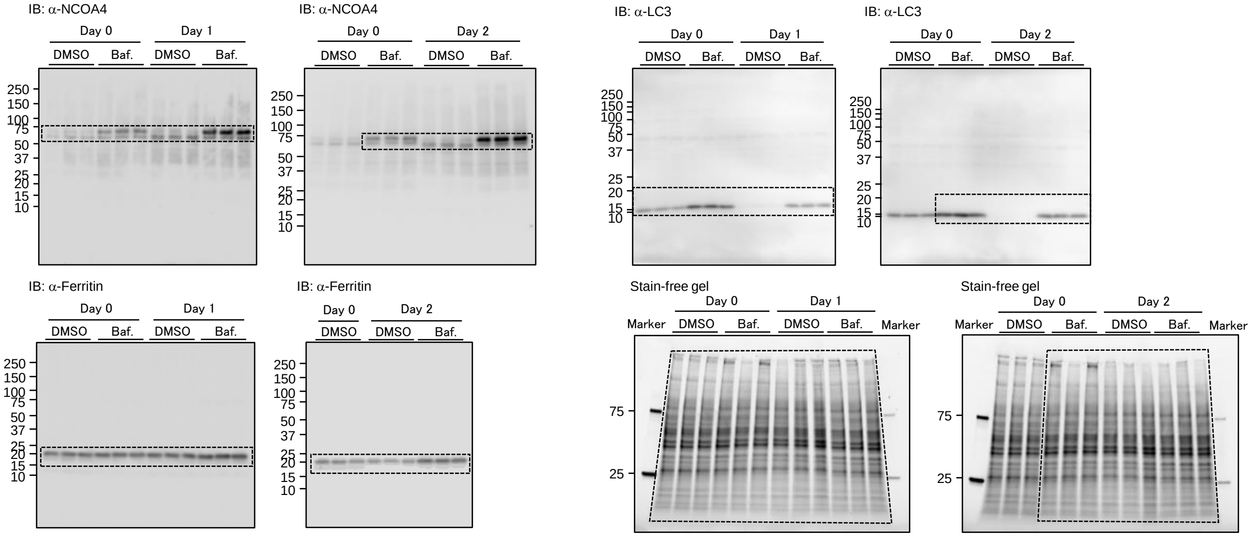

Figure 1C (top) and Supplementary Figure 1C

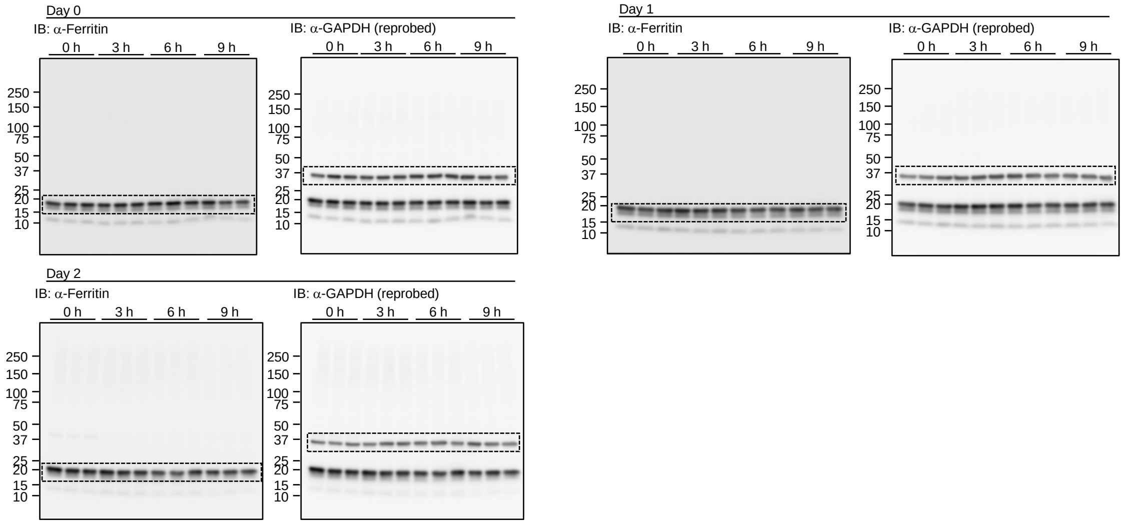

Figure 1C (bottom) and Supplementary Figure 1D

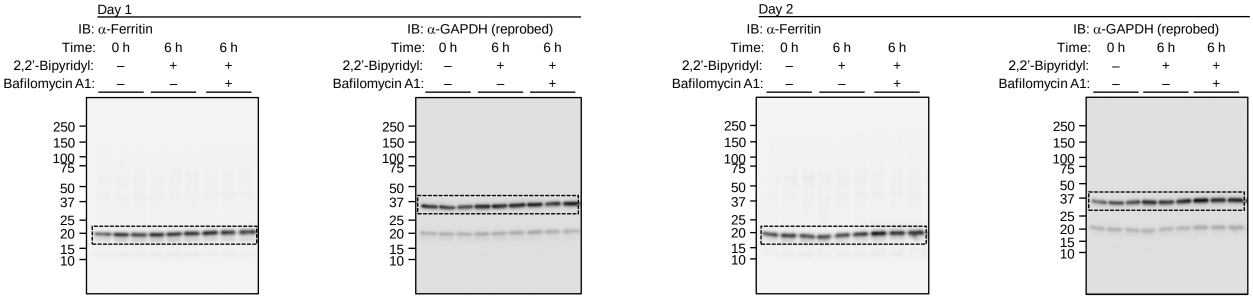

Supplementary Figure S10 continued

Figure 1H and Supplementary Figure 1F

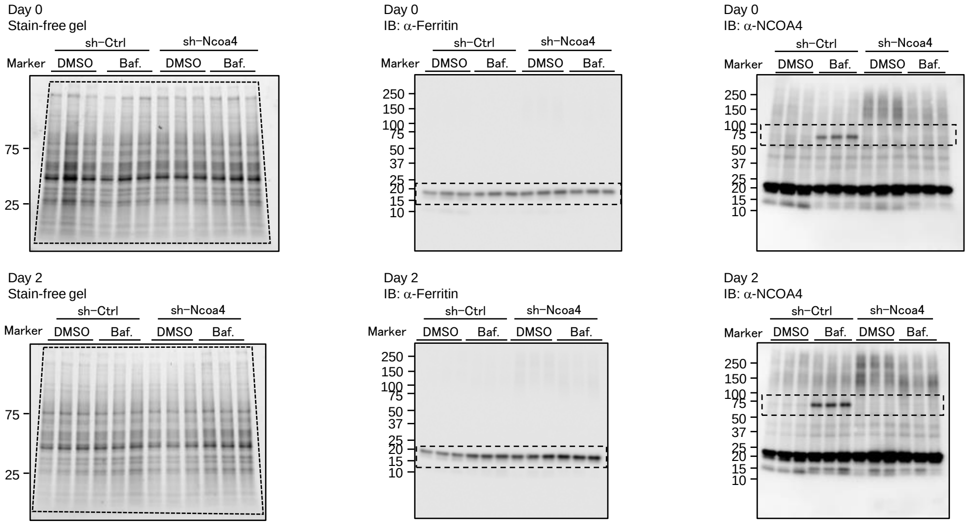

Figure 1J

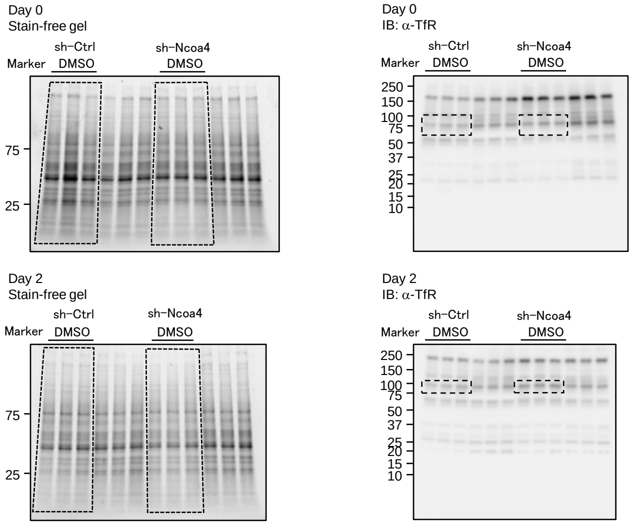

Figure 2C

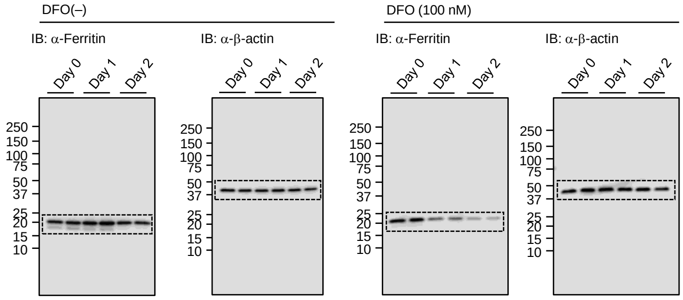

Supplementary Figure 2C

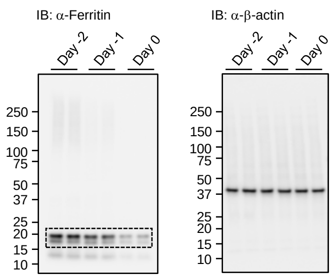

Supplementary Figure S10 continued

Figure 2G

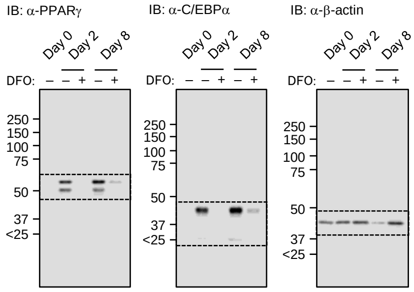

Figure 4G

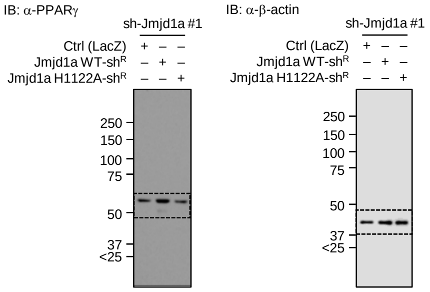

Figure 5C (top panel)

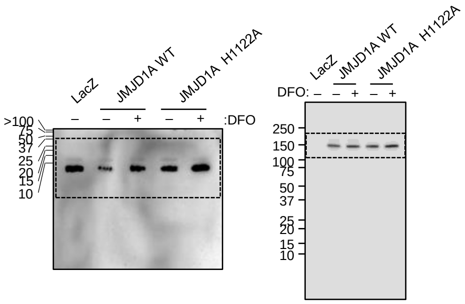

Figure 5C (bottom panel)

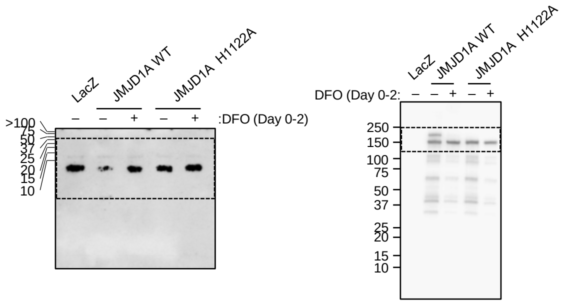

Figure 6E

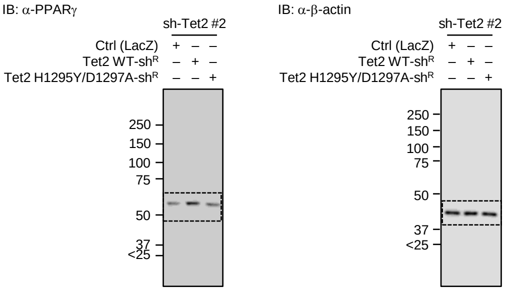

Supplementary Figure S10
continued

Figure 7B

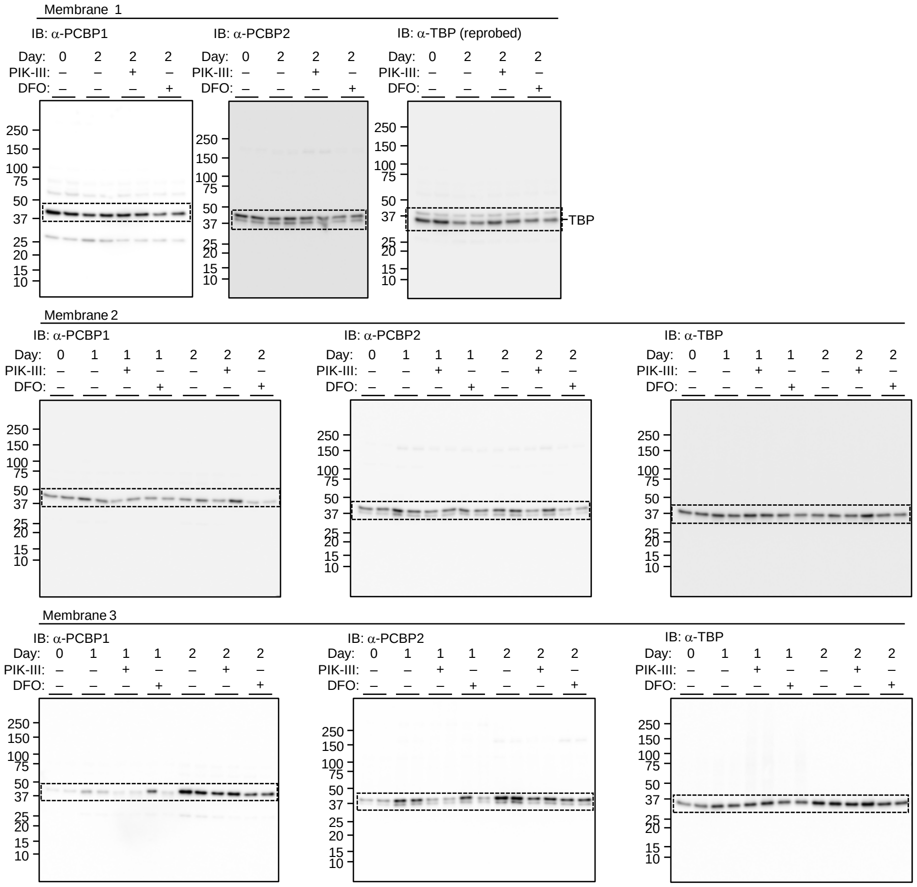

Supplementary Figure S10. Original immunoblot images.
